# Spatiotemporal Regulation of a *Legionella pneumophila* T4SS Substrate by the Metaeffector SidJ

**DOI:** 10.1371/journal.ppat.1004695

**Published:** 2015-03-16

**Authors:** Kwang Cheol Jeong, Jessica A. Sexton, Joseph P. Vogel

**Affiliations:** 1 Department of Animal Sciences & Emerging Pathogens Institute, University of Florida, Gainesville, Florida, United States of America; 2 Chemical Engineering Department, University of California, Santa Barbara, Santa Barbara, California, United States of America; 3 Department of Molecular Microbiology, Washington University School of Medicine, St. Louis, Missouri, United States of America; Tufts University, UNITED STATES

## Abstract

Modulation of host cell function is vital for intracellular pathogens to survive and replicate within host cells. Most commonly, these pathogens utilize specialized secretion systems to inject substrates (also called effector proteins) that function as toxins within host cells. Since it would be detrimental for an intracellular pathogen to immediately kill its host cell, it is essential that secreted toxins be inactivated or degraded after they have served their purpose. The pathogen *Legionella pneumophila* represents an ideal system to study interactions between toxins as it survives within host cells for approximately a day and its Dot/Icm type IVB secretion system (T4SS) injects a vast number of toxins. Previously we reported that the Dot/Icm substrates SidE, SdeA, SdeB, and SdeC (known as the SidE family of effectors) are secreted into host cells, where they localize to the cytoplasmic face of the *Legionella* containing vacuole (LCV) in the early stages of infection. SidJ, another effector that is unrelated to the SidE family, is also encoded in the *sdeC-sdeA* locus. Interestingly, while over-expression of SidE family proteins in a wild type *Legionella* strain has no effect, we found that their over-expression in a ∆*sidJ* mutant completely inhibits intracellular growth of the strain. In addition, we found expression of SidE proteins is toxic in both yeast and mammalian HEK293 cells, but this toxicity can be suppressed by co-expression of SidJ, suggesting that SidJ may modulate the function of SidE family proteins. Finally, we were able to demonstrate both *in vivo* and *in vitro* that SidJ acts on SidE proteins to mediate their disappearance from the LCV, thereby preventing lethal intoxication of host cells. Based on these findings, we propose that SidJ acts as a metaeffector to control the activity of other *Legionella* effectors.

## Author Summary

A key attribute of many pathogens is their ability to survive and replicate within eukaryotic host cells. One such pathogen, *Legionella pneumophila*, is able to grow within macrophages in the lungs, thereby causing a form of pneumonia called Legionnaires’ Disease. *L*. *pneumophila* causes disease by translocating several hundred proteins into the host cell. These proteins are typically referred to as ‘‘effectors’’, as they function as toxins to alter normal host cell function. However, since *L*. *pneumophila* remains within the host cells for approximately one day, continual poisoning of the eukaryotic cells by the bacterial effectors will result in the premature death of the host cell, thus restricting the growth of the pathogen. Previously the *L*. *pneumophila* secreted protein LubX was described as a “metaeffector”, which has been defined as an effector that acts directly on another effector to modulate its function inside the host cell. LubX accomplishes this task by directing the degradation of another effector, SidH. Here we report a second *L*. *pneumophila* metaeffector, SidJ, acts in a similar manner to neutralize SidE family effectors by removing them from the intracellular compartment that contains the bacterium. This further establishes the concept of metaeffectors, which are likely to be critical to how *Legionella* and many other pathogens cause disease.

## Introduction

*Legionella pneumophila*, the causative agent of Legionnaires' disease, is a facultative intracellular bacterial pathogen that can replicate within fresh water amoeba and mammalian alveolar macrophages [1–3]. *L*. *pneumophila* survives and replicates within host cells by inhibiting the host endocytic pathway and creating a novel replicative compartment designated as the *Legionella* containing vacuole (LCV) [4–7]. Alteration of host function is mediated by the injection of a large number of proteins into the host cell by the *L*. *pneumophila* Dot/Icm type IV secretion system (T4SS) [8–12]. However, inactivation of individual (or even combinations of) Dot/Icm substrates in genetically engineered mutant strains rarely has a strong effect on the intracellular growth of *L*. *pneumophila*, consistent with extensive functional redundancy between effectors [13–15].

One notable exception to this generalization is the *L*. *pneumophila* SuperΔP170 mutant, which exhibited a substantial growth defect in the amoebae *Acanthamoeba castellanii* [16]. The SuperΔP170 was constructed while studying a locus that encodes multiple Dot/Icm substrates [16] and consists of two deletions: the first removes five adjacent genes (*sdeC*, *lpg2154*, *sidJ*, *sdeB*, and *sdeA*) and the second deletes the unlinked gene *sidE*. Four of the encoded proteins, SidE, SdeC, SdeB and SdeA, share extensive homology with each other and are all ∼170 kDa in size, thus they have been referred to as “P170s” [16]. In addition, they are called the “SidE family”, as SidE was the founding member of this related group of proteins [17]. The SidE proteins are Dot/Icm substrates that are translocated into the host cell and reside on the cytoplasmic face of the LCV (*Legionella* containing vacuole) [16], although their molecular function is not known. As the intracellular growth defect of the SuperΔP170 mutant could be complemented by expression of just one SidE family protein, SdeA, it was proposed that the SidE-like proteins were functionally redundant and the other two genes, *lpg2154* and *sidJ*, must be dispensable for growth within host cells [16]. However, subsequently it was shown that inactivation of *sidJ* alone conferred an intracellular growth defect on *L*. *pneumophila* [18], suggesting the situation is more complicated than initially perceived.

Consistent with this observation is the increasingly appreciated paradigm in pathogenesis that secreted effectors are often subjected to spatiotemporal regulation and that there can be a complex interplay between the functions of different effectors. For example, the *Salmonella* T3SS substrates SopE and SptP, which possess opposing biochemical activities, act at different stage of infection to first induce bacterial uptake and then to down-modulate this effect in order to prevent host cell death [19]. Similarly, the *Legionella pneumophila* Dot/Icm T4SS effectors SidM/DrrA and LepB exhibit opposing functions. SidM/DrrA recruits and activates Rab1 to mediate fusion of ER microsomes with the LCV (*Legionella* containing vacuole). At later points, LepB inactivates Rab1 resulting in the removal of the GTPase from the LCV [20–22]. A third example is represented by the *L*. *pneumophila* effectors SidH and LubX. SidH is a homolog of the effector SdhA, which is required to maintain the integrity of the LCV [23,24]. LubX is a member of the U-box family of E3 ubiquitin ligases and functions as a metaeffector to inactivate SidH by promoting its proteolysis [25].

Due to their genetic proximity, the surprising phenotype of the Δ*sidJ* mutant, and the existing precedents of complex interplay between other *L*. *pneumophila* secreted substrates, we hypothesized that there may be a connection between the SidE proteins and SidJ. To test this hypothesis, we examined the phenotypes of a strain lacking just the four SidE proteins (Δ*sdeC* Δ*sdeB* Δ*sdeA* Δ*sidE*) and of an individual Δ*sidJ* mutant and discovered that overexpression of an individual SidE family protein in the absence of SidJ is toxic to host cells. This result, and the experiments that followed, have led to a model wherein SidJ functions as a metaeffector to regulate the activity of the SidE family of toxins.

## Results

### SidJ and the SidE family are required for optimal intracellular growth of *L*. *pneumophila*

**Fig 1:**
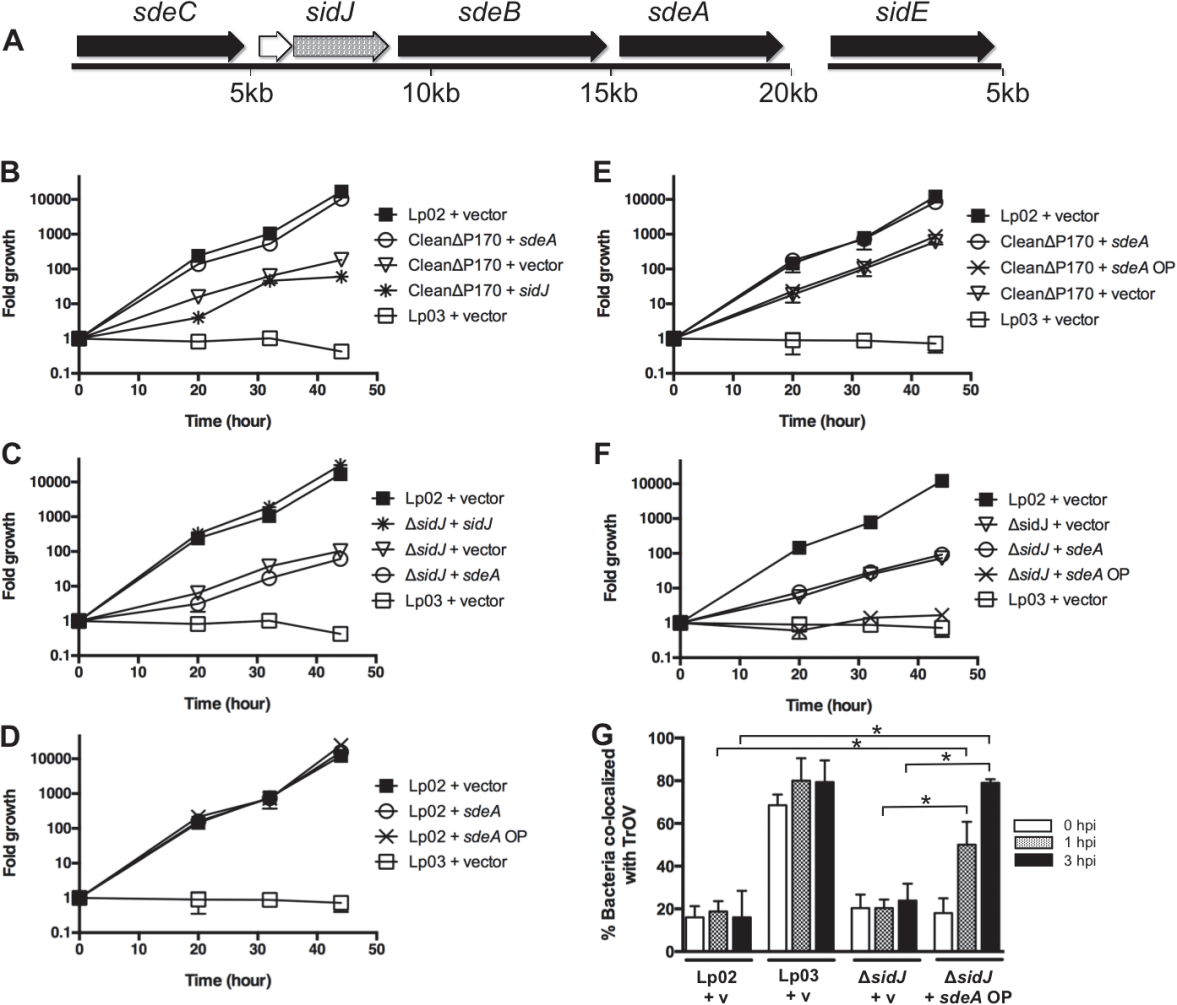
Genetic analysis of the SidE family and SidJ. (A) *sdeC-sdeA* locus includes five genes (*sdeC*, *lpg2154*, *sidJ*, *sdeB*, and *sdeA*), whereas the *sidE* gene is located at a separate location. The SidE family consists of the related proteins SidE, SdeC, SdeB, and SdeA (shown with black arrows). (B-F) Replication of various *L*. *pneumophila* strains in *A*. *castellanii* was determined at the indicated time points post infection and expressed as fold growth. (B) Suppression of the growth defect of the CleanΔP170 mutant by expression of low amounts of SdeA. (C) Complementation of the Δ*sidJ* mutant by expression of SidJ. (D-F) Overexpression of SdeA does not inhibit the growth of the wild type strain Lp02 (D) or the CleanΔP170 mutant (E) but does inhibit the replication of the Δ*sidJ* mutant (F). (G) Overproduction of SdeA causes *Legionella* to traffic into the endocytic pathway of amoebae. *A*. *castellanii* pre-incubated with Texas Red Ovalbumin (TrOV), which labels their endocytic pathway and vacuole, were infected with Lp02, a *dotA* mutant (Lp03) and a Δ*sidJ* mutant containing vector (v) or a plasmid over-expressing SdeA (*sdeA* OP). The percent of bacteria that co-localized with TrOV was quantitated at 0, 1, and 3 hours post infection. Data are means ± SEM of three independent experiments. Approximately 100 bacteria were scored per condition and asterisks indicate statistical difference (*P*<0.05).

The *sdeC-sdeA* locus encodes SdeC, Lpg2154, SidJ, SdeB, and SdeA (Fig. 1A). A related protein, SidE, is encoded at a separate site on the chromosome. SidE, SdeC, SdeB, and SdeA are each ∼170 kDa in size, share greater than 40% identity to each other, and are substrates of the *L*. *pneumophila* Dot/Icm T4SS (S1 Fig.) [16,17]. The *sdeC-sdeA* locus also encodes another Dot/Icm substrate, SidJ, which has no homology to the SidE family (S1 Fig.). Although the *L*. *pneumophila* strain Philadelphia I encodes a homolog to SidJ, SdjA (Lpg2508), it was previously shown to be dispensable for virulence and therefore was not characterized further [18].

To examine the connection between SidJ and the SidE family of proteins, we constructed two additional mutant strains. One mutant lacked only *sidJ* and the other mutant contained deletions in each of the four *sidE*-related genes, which we refer to as the “CleanΔP170” mutant. We then compared the intracellular replication properties of the Δ*sidJ*, the CleanΔP170, and the original SuperΔP170 mutant [16], which does not express SidJ or any member of the SidE family. Growth was assayed by infecting the amoebae *A*. *castellanii* for various amounts of time, lysing the cells and determining the fold bacterial growth based on the number of CFUs (colony forming units). In this assay, a wild-type strain of *L*. *pneumophila* was able to replicate greater than 1000-fold in forty-four hours whereas a T4SS-deficient strain, Lp03, was unable to grow (Fig. 1B). In contrast, the CleanΔP170 strain exhibited a 100-fold growth defect similar to that previously observed for the SuperΔP170 mutant (Figs. 1B and S2). Likewise, a strain lacking *sidJ* exhibited a similar intermediate intracellular replication deficiency (Fig. 1C) [18]. The CleanΔP170 growth defect could be complemented by expression of just one SidE family protein, SdeA, consistent with the redundancy previously observed between members of this family (Fig. 1B) [16]. As expected, the replication defect of the CleanΔP170 mutant could not be complemented by expression of SidJ from a plasmid as this strain already makes SidJ (Fig. 1B). Similarly, the strain lacking *sidJ* could be complemented by expression of *sidJ*, but not by *sdeA* (Fig. 1C).

Taken together, these data suggest that expression of SidJ and at least one member of the SidE family is required for virulence of *L*. *pneumophila* within the environmental host *A*. *castellanii*. But this raised the question of how the original SuperΔP170 mutant strain, which is missing all four members of the SidE family and SidJ, could be fully complemented by expression of only SdeA (S2 Fig.) [16]. We hypothesized that this discordance might be related to expression levels from the *sdeA* expression plasmid. However, the *sdeA* complementing clone, pJB3556, did not synthesize aberrant amounts of SdeA and instead made a similar amount to what is normally expressed in *Legionella* (S3 Fig.).

Although pJB3556 expresses the appropriate level of SdeA in a cell, it is worth noting that this represents only a portion of the total amount of SidE family proteins expressed in a wild type cell. Therefore, we assayed the effect of expressing higher amounts of SdeA from a new complementing clone, SdeA OP (SdeA “over production”) (S3 Fig.). Expression of SdeA from this new construct had no effect on the growth of the wild type strain Lp02 (Fig. 1D) or the CleanΔP170 mutant (Fig. 1E). On the other hand, over-expression of SdeA remarkably completely inhibited the growth of the Δ*sidJ* mutant to levels similar to that of the T4SS-deficient *dotA* mutant (Fig. 1F). This extent of inhibition was shared with the SuperΔP170 mutant, which also does not express SidJ (S2 Fig.). Thus, inhibition of replication by SdeA over-production occurs only in strains lacking *sidJ*. Interestingly, this inhibitory effect appears to be specific to *Legionella* virulence, as over-expression of SdeA in the Δ*sidJ* mutant results in increased mis-targeting of *Legionella* to a late endocytic/lysosomal compartment (Fig. 1G). In summary, the absence of either the SidE family or SidJ results in diminished growth of *L*. *pneumophila* within amoebae. Furthermore, the partial growth defect of a Δ*sidJ* strain, but not of a wild type strain, can be exacerbated by the over-expression of SdeA, thus establishing a link between SidJ and the SidE family.

### SidJ and SdeA do not affect each other’s secretion into host cells

One possible connection between SidJ and SdeA is that they might modulate each other’s secretion into host cells. To test this hypothesis, we measured the export of the reporter proteins CyaA-SidJ and CyaA-SdeA in different genetic backgrounds. Successful export of *Bordetella pertussis* CyaA fusions into the host cell cytoplasm is measured by increased cAMP production, since this version of CyaA is activated when it is bound by eukaryotic calmodulin [26,27]. Expression of either CyaA-SidJ or CyaA-SdeA in the wild-type strain Lp02 generated a large increase in host cell cAMP as compared to expression in T4SS-deficient Lp03 cells, mock infected cells, or Lp02 expressing only CyaA (S4 Fig.). As previously observed [16,28], SdeA secretion was strongly dependent on the type IV adaptor IcmS whereas SidJ secretion was only partially dependent. However, export of SdeA was not affected by the absence of *sidJ* nor was SidJ secretion diminished in a strain that did not express any of the SidE family (S4 Fig.). Therefore, SidJ does not appear to regulate the secretion of SidE family members and SidE proteins do not affect the export of SidJ, suggesting that the molecular connection between SidJ and SidE family members likely occurs within host cells.

### SdeA toxicity in eukaryotic cells can be suppressed by SidJ

**Fig 2:**
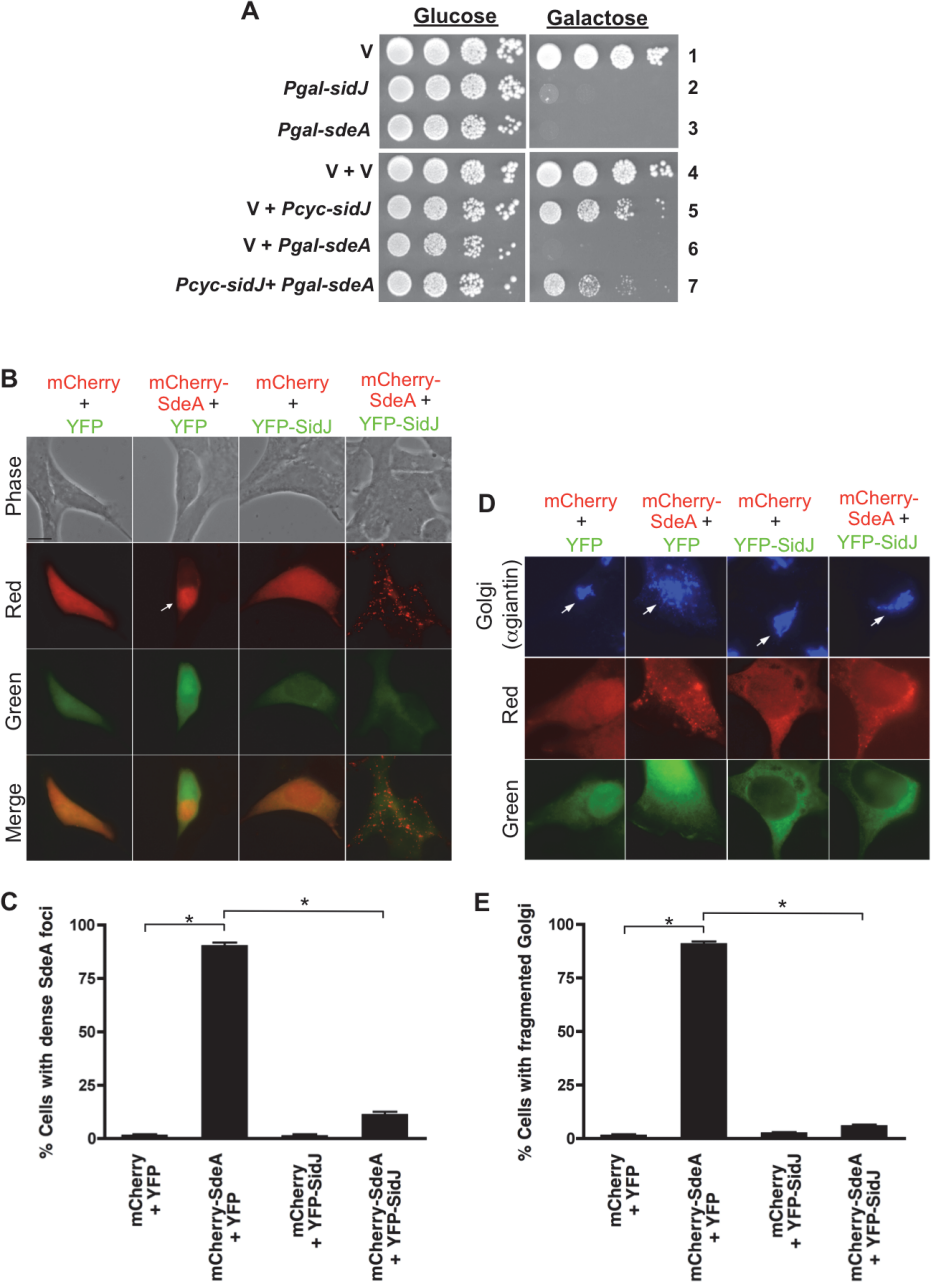
SidJ suppresses SdeA toxicity in yeast and mammalian cells. (A) Tenfold serial dilution of yeast strains expressing vector (v), P*gal*-*sidJ*, P*gal*-*sdeA* or P*cyc*-*sidJ* were spotted and grown on selective plates containing glucose (repressing conditions) or galactose (inducing conditions). (B and C) Localization of SdeA and SidJ in HEK293 cells as observed by transfecting cells for 40-hours with mCherry, YFP, mCherry-SdeA and YFP-SidJ. Channels used to acquire images are indicated on the left of each row and merged images are shown in the bottom row. Arrow indicates a cell containing a dense mCherry-SdeA foci. Bar, 10 μm. (C) Scoring data of cells with dense SdeA loci observed in (B). (D) Shorter transfection time (20-hour) reveals mCherry-SdeA-induced Golgi fragmentation in HEK293 cells. Cells were fixed and stained with anti-giantin (Golgi marker). Bar, 10 μm. Arrows indicate Golgi. (E) Scoring data of Golgi fragmentation in (D). Data are means ± SEM of three independent experiments. Approximately 100 cells/condition were counted and asterisks indicate statistical difference (*P*<0.05).

Since over-expression of SdeA is toxic to amoebae in the absence of SidJ, we chose to explore this phenomenon in more detail within the model eukaryote *Saccharomyces cereviseae* [29,30]. We began these yeast studies by expressing SdeA and SidJ under the control of the strong, regulated *Pgal* promoter. Yeast cells transformed with *Pgal* vectors carrying *sdeA* or *sidJ* were grown in the presence of glucose, diluted, and spotted onto selective media containing glucose (repressing conditions) or galactose (inducing conditions). Galactose-induced expression of either SdeA or SidJ was extremely toxic to yeast cells (Fig. 2A, rows 2–3) consistent with previous results [29,30]. To further evaluate this toxicity, we expressed each protein at lower levels using the constitutively expressed, weak promoter *Pcyc*. Expression of *sidJ* under the weaker promoter had only a subtle effect on yeast (Fig. 2A, row 5) whereas it was not possible to construct a yeast strain harboring *Pcyc*-*sdeA*, presumably due to SdeA-mediated toxicity. Strikingly, expression of low amounts of SidJ (*Pcyc*-*sidJ*) was able to partially suppress the toxicity caused by expression of high amounts of SdeA (*Pgal*-*sdeA*) (Fig. 2A, row 7).

We then attempted to recapitulate this result in mammalian cells. Transient transfection of HEK293 cells for 40 hours with mCherry-SdeA resulted in the protein localizing in dense foci in ∼90% of the cells (Fig. 2B and 2C). This result was specific to the SdeA fusion as expression of only mCherry resulted in diffuse, cytoplasmic staining. The longer mCherry-SdeA was expressed in cells, the more toxic it became eventually causing the cells to round up (S5 Fig.). In contrast, expression of YFP-SidJ was not toxic and the protein localized diffusely in the cytoplasm similar to YFP alone (Fig. 2B and 2C). Remarkably, the accumulation and toxicity caused by mCherry-SdeA expression was suppressed by co-transfection with YFP-SidJ resulting in dispersal of mCherry-SdeA into smaller foci (Fig. 2B and 2C).

To further examine SdeA toxicity in mammalian cells, mCherry-SdeA was expressed for a shorter amount of time (20 hours) and the cells were analyzed by fluorescence microscopy using markers to detect the endoplasmic reticulum, mitochondria, Golgi, endosome/lysosome, actin, and tubulin. Shorter expression of mCherry-SdeA only affected the Golgi resulting in its fragmentation (Fig. 2D-2E). In contrast, expression of mCherry alone, YFP alone, or YFP-SidJ had no effect on the Golgi. Similar to the redistribution of SdeA, YFP-SidJ was able to suppress the Golgi fragmentation caused by mCherry-SdeA (Fig. 2D and 2E). In summary, SdeA expression was toxic to both yeast and mammalian cells and SdeA-toxicity could be suppressed by co-expression of SidJ, implying that SidJ may regulate SdeA function.

### SidJ modulates SdeA localization on *Legionella* containing vacuoles

A number of Dot/Icm substrates localize to the outside of the *Legionella* containing vacuole (LCV) at various stages of infection, including members of the SidE family [16,17,22,31]. For example, the substrate SidM/DrrA associates with the LCV early during infection but cannot be detected at later time points [22]. In contrast, the substrate LepB has limited association with the LCV at early points but displays increased co-localization over time. Similar to SidM/DrrA, SidE family proteins can be detected in proximity to the LCV early on but then disappear at later points during infection [16].

**Fig 3:**
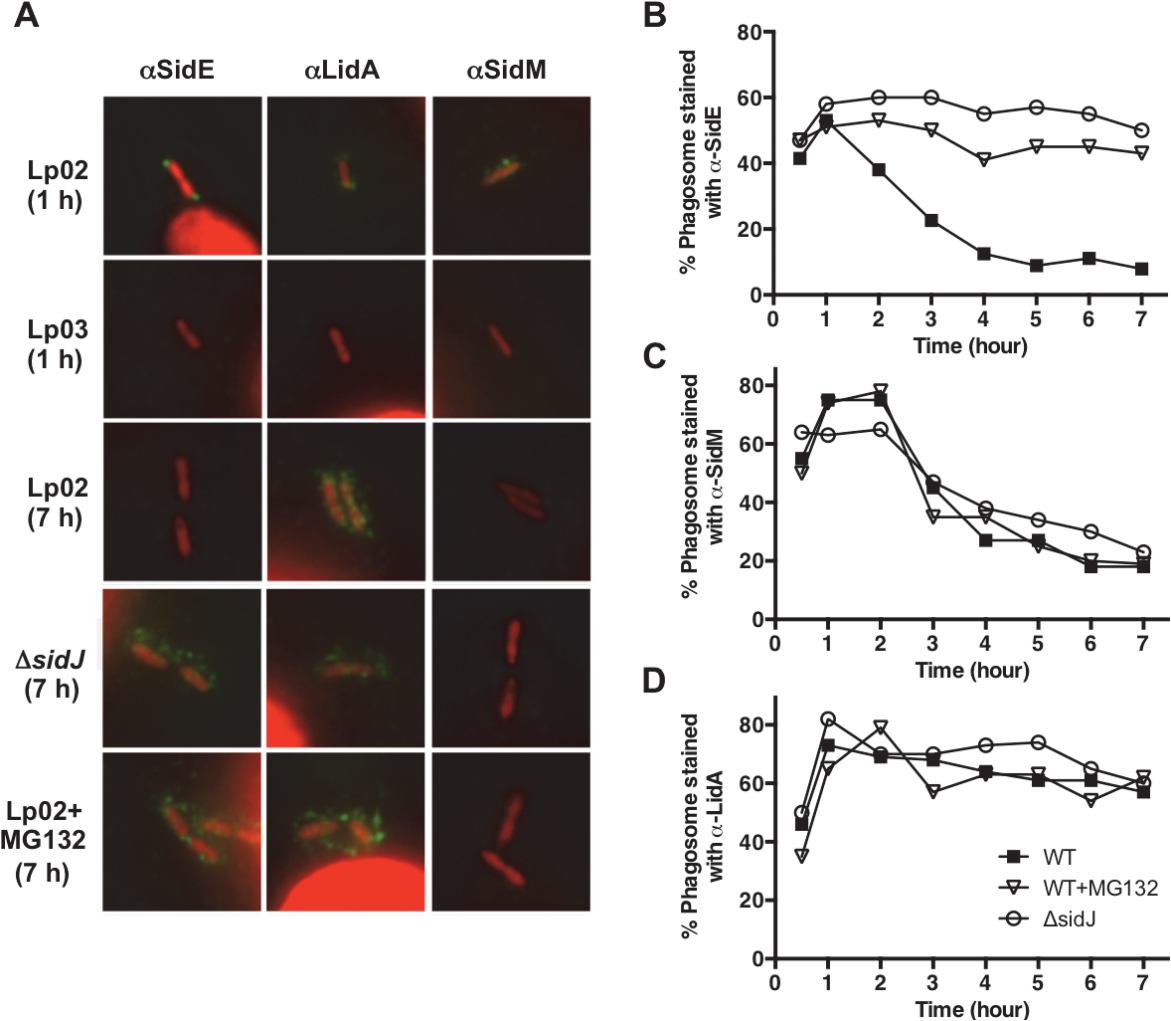
SidJ mediated the disappearance of SidE proteins from the LCV at later time points of infection. (A) BMMs were infected with the wild-type strain Lp02, the T4SS-deficient strain Lp03 or the Δ*sidJ* mutant, fixed, and stained with antibodies specific for the SidE family, LidA, or SidM (green). DNA (*L*. *pneumophila* and host nuclei) was stained with propidium iodide (red). Representative images are shown for 1 hour or 7-hour infections. Cells were treated with MG132 (10 μM) for 15 min prior to infection as needed. (B-D) Co-localization of SidE, SidM, and LidA with the LCV were scored and recorded as a percentage. Percentage of co-localization is plotted over time for SidE proteins (B), SidM (C), and LidA (D) for the wild-type strain Lp02 (filled squares), Lp02 + MG132 (open triangles), and the Δ*sidJ* mutant (open squares). Approximately 75 LCVs were counted for each Dot/Icm substrate at each point. Two independent experiments were conducted and the data is representative of both.

Based on these results, we hypothesized that SidJ may modulate SidE family association with the LCV as a means of regulating their activity. We began by confirming the observation that SidE family proteins can be detected on the LCV only at the initial stages of infection [16]. Bone marrow macrophages (BMMs) were infected for increasing amounts of time with the wild-type *L*. *pneumophila* strain Lp02, the T4SS-deficient strain Lp03, and the Δ*sidJ* mutant. The cells were fixed and stained with antibodies specific for three Dot/Icm substrates including the SidE family [16], SidM/DrrA, and a protein known to remain on the LCV, LidA [31]. As shown in Fig. 3, SidE staining could be detected on wild-type LCVs adjacent to the bacterial poles at 1-hour post infection. The detected SidE signal at 1 hour represented secreted protein, as it was not detected in Lp03-infected macrophages. As previously observed [16], SidE staining decreased as the infection proceeded resulting in less than 10% co-localization with the LCV after 4 hours (Fig. 3B). SidM/DrrA exhibited a similar pattern of localization over time (Fig. 3C) [22]. In contrast to SidE and SidM/DrrA, the secreted effector LidA was retained on the LCV throughout the infection (Fig. 3D), thus demonstrating that the disappearance of SidE and SidM/DrrA is not a general phenomenon.

To test our hypothesis that SidJ might mediate SidE family localization, we examined the intracellular position of the three Dot/Icm substrates when secreted from a Δ*sidJ* mutant. The absence of SidJ had no effect on the location of SidM/DrrA or LidA (Fig. 3). However, the localization of SidE was significantly altered in the Δ*sidJ* strain in a time-dependent manner resulting in retention of SidE on the LCV at later time points of infection (Fig. 3B). The disappearance of SidE proteins from the LCV in a wild-type infection could be due to their degradation and/or their dissociation from the *L*. *pneumophila* phagosome. To test these possibilities, we examined the presence of LCV-associated SidE proteins during an infection where the host cells were first treated with the proteasome inhibitor MG132. Interestingly, MG132 treatment resulted in a significant increase in the amount of retained SidE proteins at later time points (Fig. 3B), similar to that seen with the Δ*sidJ* mutant. In contrast, MG132 had no effect on SidM localization (Fig. 3C), thus demonstrating specificity. In summary, the disappearance of SidE proteins from the LCV at later points of infection is dependent on both SidJ and the proteasome.

### SidE disappearance from the LCV is not due to general proteolysis of the protein

Based on the MG132 result, it is possible that SidJ removes SidE family proteins from the LCV via induced proteolysis, similar to the action of the metaeffector LubX on the substrate SidH [25]. To test this theory, we developed a method using the detergent digitonin to measure the total amount of secreted SidE family proteins. Digitonin extracts proteins from mammalian membranes without disrupting bacterial membranes due to the absence of cholesterol in the latter membranes. We then proceeded to infect the human monocytic cell line U937 with wild-type *Legionella*, gently lysed the cells using a dounce homogenizer and removed unbroken host cells via low speed centrifugation. This generated a post-nuclear supernatant (PNS) fraction that was then separated by a high-speed centrifugation step into a pellet fraction, containing host organelles and the LCV, and a soluble cytoplasmic fraction.

**Fig 4:**
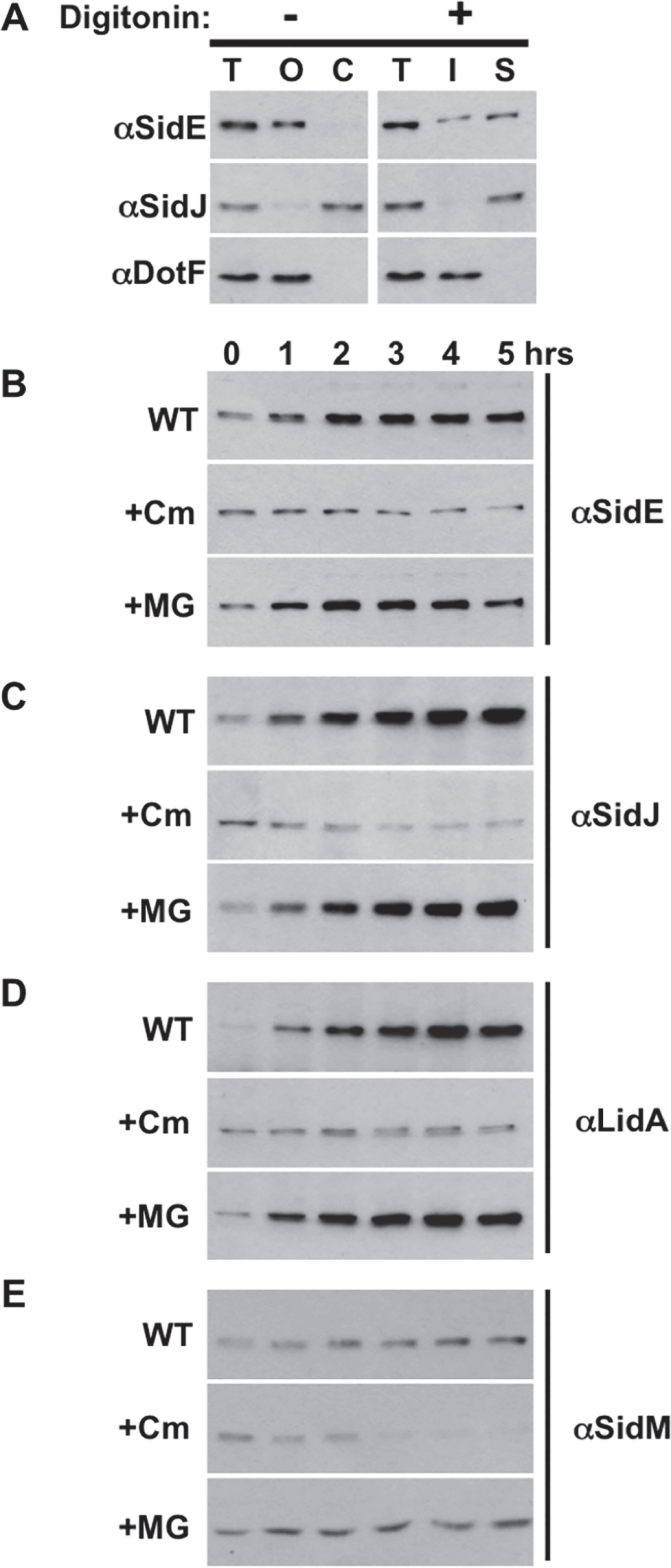
SidE proteins are not degraded during infection. (A) Assay demonstrating secretion of Dot/Icm substrates SidE and SidJ. U937 cells were infected with wild-type *Legionella*, lysed by douncing, and post nuclear supernatant fractions (PNS) were prepared. Samples were processed in the absence (left column) or the presence of digitonin (right column). Fractions include total (T), organelles and LCV (O), cytoplasm (C), digitonin insoluble (I) and digitonin soluble (S), which contains secreted proteins. Fractions were analyzed by Western blot using antibodies specific for the indicated proteins. (B-E) U937 cells were infected with wild-type *Legionella* for 1 hour, washed, and the infection was allowed to proceed for the indicated times in the absence (WT) or presence of chloramphenicol (Cm) or MG132 (MG). The cells were processed as above including dounce lysis and digitonin treatment followed by centrifugation. The digitonin soluble data is shown for SidE (B), SidJ (C), LidA (D), and SidM (E) and is representative of three experiments.

In the absence of digitonin, the majority of SidE-like proteins co-localized in the pellet fraction with DotF, an inner membrane protein of the Dot/Icm T4SS that was used as a marker for the LCV (Fig. 4A). In contrast, SidJ could be detected in the soluble fraction without digitonin, indicating that the majority of the protein was secreted into the host cytoplasm and not retained on the LCV (Fig. 4A). Inclusion of digitonin in the reaction resulted in the solubilization of a large percentage of the SidE proteins, indicating they were secreted into the host cell. DotF could not be extracted and therefore was retained within the digitonin-resistant, bacterial cell wall (Fig. 4A).

Using this PNS/digitonin assay, we measured the levels of secreted SidE and SidJ proteins by Western analysis. Rather than observing decreased quantity of digitonin-soluble SidE proteins consistent with proteolysis, their amounts actually increased over the first 5 hours of the infection (Fig. 4B, WT). The levels of secreted SidJ and LidA were elevated in a similar fashion, although the amount of SidM/DrrA did not significantly increase over time (Fig. 4B and 4C, WT). In order to more carefully examine if any of the initial, secreted protein was degraded, we examined the levels of the proteins after inhibition of protein synthesis using the antibiotic chloramphenicol. The initial wave of secreted SidE, SidJ, and LidA remained fairly stable in the presence of chloramphenicol (Fig. 4, +Cm). In contrast, chloramphenicol-treatment did affect the levels of SidM/DrrA as the infection proceeded, suggesting that continual secretion of SidM/DrrA protein was necessary to maintain the levels of the protein over time (Fig. 4E, +Cm).

Treatment with the proteasome inhibitor MG132 resulted in a slight stabilization of SidM/DrrA consistent with the decreased amounts of SidM in the presence of chloramphenicol being due to proteolysis by the proteasome (Fig. 4E, +MG). In contrast, MG132 surprisingly did not alter the levels of SidE proteins (Fig. 4B, +MG), indicating they were not subject to proteasomal degradation. This result was unexpected based on our previous data showing MG132 treatment prevented the disappearance of SidE from the LCV (Fig. 3). Therefore, the situation must be more complex than initially anticipated. For example, there could be two populations of SidE proteins within the cell, a small portion on the LCV and a distinct population located elsewhere, and SidJ mediates the proteasomal-degradation of only LCV-associated SidE proteins.

### SidE disappears from the LCV and co-localizes with fractions containing host organelles at later time points of infection

**Fig 5:**
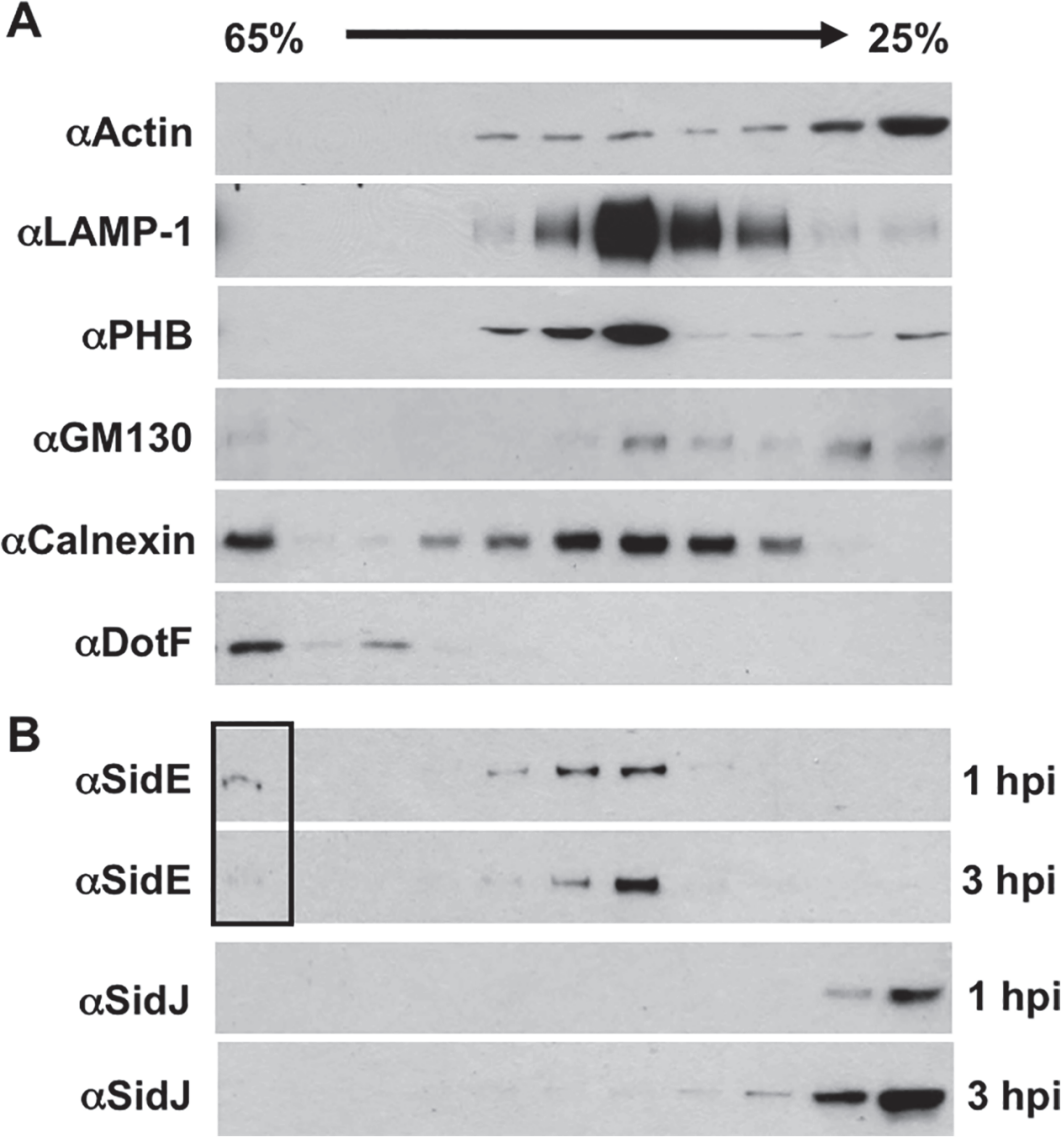
SidE proteins removed from the LCV associate with host organelles. U937 cells were infected with wild-type *Legionella* for 1 hour or 3 hours, lysed by douncing, separated by sucrose gradient and analyzed by Western blot. (A) Western blots includes markers for various cell compartments including: actin (cytoplasm), LAMP-1 (endocytic compartments), PHB (mitochondria), GM130 (Golgi), calnexin (ER), and DotF (LCV). (B) Westerns for SidE proteins and SidJ at both 1 and 3 hour post infection (hpi). The box highlights the altered levels of SidE proteins in the LCV fraction as the infection progresses.

To test this hypothesis, we separated the LCV from other cell organelles using discontinuous sucrose gradient analysis. Using this method, we were able to obtain fractions enriched for the cytoplasm (actin), endosomes/lysosomes (LAMP-1), mitochondria (PHB), Golgi (GM130), ER (calnexin), and the LCV (DotF) (Fig. 5A). We then examined the location of SidE proteins and SidJ at 1 hpi (hour post infection) and 3 hpi. Consistent with our immunofluoresence data in Fig. 3, we were able to detect SidE proteins that co-localized with the LCV at 1 hpi (Fig. 5B). However, we also observed a large amount of SidE proteins in fractions that contained host organelles, including endosomes/lysosomes, mitochondria and ER (Fig. 5). Strikingly, at 3-hours post infection there was a significant depletion of SidE proteins in the LCV fraction and instead there was an enrichment of these proteins in the organelle fractions distinct from the peak cytoplasmic fractions containing actin (Fig. 5A). In contrast to SidE proteins, SidJ was found in the cytoplasmic fractions at both 1 and 3-hours post infection (Fig. 5B). Taken together, our data suggests that the SidJ and proteasome-dependent removal of SidE proteins from the LCV is due to localized degradation of protein.

### SidJ directly mediates the disappearance of SidE proteins from the LCV

**Fig 6:**
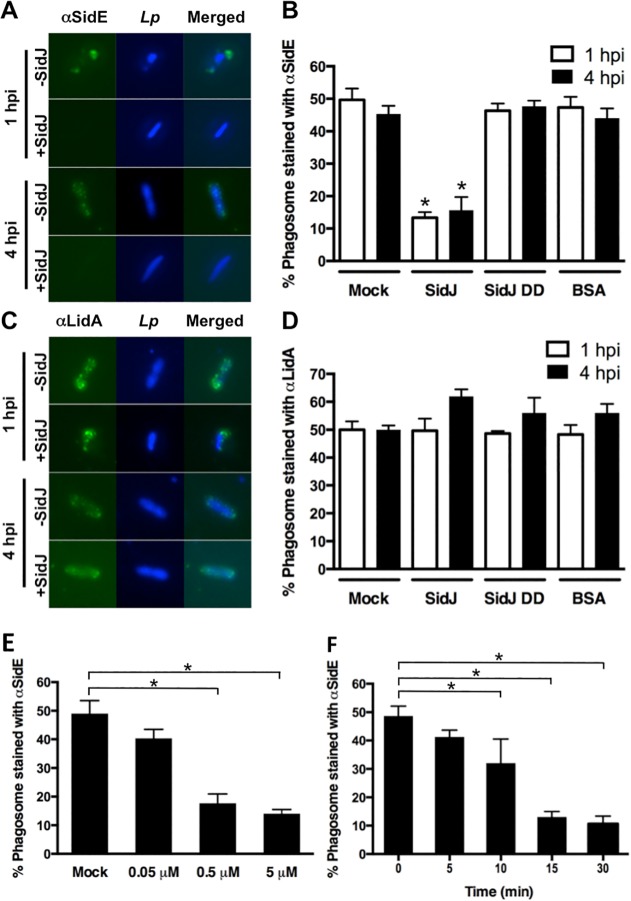
*In vitro* assay for SidJ removal of SidE proteins from the LCV. (A-D) BMMs were infected with a Δ*sidJ* mutant for the indicated time, lysed by gentle douncing, unbroken cells removed by centrifugation, and the PNS was incubated with 0.5 μM of purified SidJ, SidJ DD mutant or BSA. The reactions were stopped by addition of paraformaldehyde. The fixed cells were then stained with anti-SidE (A) or LidA (C) antibody. Representative images are shown with SidE and LidA in green and bacteria in blue. (B and D) Phagosomes were scored for co-localization with SidE proteins (B) or LidA (D). Removal of SidE proteins from the LCV is dependent on the concentration of SidJ (E) and the time of incubation (F). Data are means ± SEM of three independent experiments. Approximately 75 LCVs were counted for each reaction and asterisks indicate statistical difference among treatments in both B and D and in samples compared to the mock controls in both E and F, (*P*<0.05).

Although the removal of SidE proteins from the LCV requires SidJ, it is possible that this effect is indirect and perhaps due to maturation of the LCV over time. Therefore, we developed an assay to test if purified SidJ protein could remove SidE proteins from LCVs *in vitro*. This assay involved isolating post nuclear supernatants (PNSs) from bone marrow-derived macrophages (BMMs) infected for 1 hour or 4 hours with the *L*. *pneumophila* Δ*sidJ* mutant (Fig. 6). The PNSs were mock treated or incubated with purified wild-type SidJ, a SidJ mutant (SidJ DD), or BSA as a negative control. The SidJ DD mutant contains mutations in two conserved aspartate residues (D542A D545A) (S6B Fig.), was fortuitously identified based on a fallacious lead using a protein fold recognition server (Phyre), but resulted in a protein that nevertheless failed to complement the intracellular growth defect of a Δ*sidJ* mutant (S6C Fig.). As shown in Fig. 6A and 6B (mock), SidE proteins remained associated with the polar sites of the *Legionella* phagosome in the absence of SidJ at both 1-hour and 4-hour post infection. Addition of purified wild-type SidJ to the PNS displaced the SidE proteins from LCVs obtained from either the 1-hour or the 4-hour infection. In contrast, inclusion of an equivalent amount of the non-functional SidJ mutant, SidJ DD, or BSA had no effect on SidE co-localization with the LCV (Fig. 6A and 6B). The disappearance of SidE was specific as addition of SidJ did not cause removal of LidA from the LCV (Fig. 6C and 6D). SidJ-mediated removal of SidE was both concentration (Fig. 6E) and time-dependent (Fig. 6F) consistent with a catalytic mechanism.

The ability of SidJ to remove SidE proteins from the LCV *in vitro* using lysates prepared from either 1 hour or 4 hours infections indicates that their absence is not simply due to LCV maturation but rather is a direct consequence of SidJ action. In addition, concentration-dependence of the reaction is consistent with SidJ accumulation in the host cell cytoplasm at later time points of infection (Fig. 4). In summary, these data suggest that the SidE proteins function as toxins during early stages of infection and that SidJ inactivates them by mediating their active removal from the *Legionella*-containing vacuole.

## Discussion

The pathogen *L*. *pneumophila* serves as an excellent model system to study the interactions between secreted effector proteins, as it exports between 200–300 substrates via its Dot/Icm T4SS [32]. In this study, we examined the relationship between the *L*. *pneumophila* Dot/Icm substrates SidJ and the SidE family. Six lines of evidence support a functional connection between these proteins. First, SidJ and SdeC, SdeB, and SdeA are all expressed from the same locus (Fig. 1). Second, the SuperΔP170 mutant, the CleanΔP170 mutant, and the Δ*sidJ* mutant each have a similar growth defect within host cells (Figs. 1 and S2). Third, expression of low levels of SdeA was able to complement/suppress the intracellular growth defect of the SuperΔP170 mutant, which lacks both the *sidE* family genes and *sidJ* (Fig. 1). Fourth, overexpression of SdeA was detrimental for the growth of only strains lacking *sidJ* (Fig. 1). Fifth, SdeA toxicity to yeast and mammalian host cells could be suppressed by co-expression of SidJ (Fig. 2). Sixth, SidJ is able to promote the disappearance of SidE proteins from the LCV at later points of infection (Fig. 3) and this could be reproduced in an *in vitro* assay using purified SidJ (Fig. 6). Based on these results, we propose that the *L*. *pneumophila* SidJ protein functions as a metaeffector to regulate the activity of the SidE protein family.

The concept that intracellular pathogens must regulate the activity of their secreted effectors during an infection is not surprising, as unregulated toxin activity would lead to the premature demise of the host cell. One method of regulation might entail the spatiotemporal delivery and/or control of substrates with opposing activities. For example, *Salmonella’s* SopE and SptP toxins act antagonistically to activate and inactivate Rho-family GTPases CDC42 and Rac1 at different times of infection via a combination of differential activity and temporal stability [19]. Likewise *L*. *pneumophila* regulates the activity of Rab1 by using a GEF (SidM/DrrA) and a GAP (LepB) [20–22]. In addition to this general mode of GTPase regulation, *L*. *pneumophila* is able to stabilize Rab1 in an active form using ampylation by the effector SidM/DrrA and then reverse the effect via de-ampylation by SidD [33–36]. *Legionella* also employs two additional effectors with opposing activities, AnkX and Lem3/Lpg0696, to inactivate and then release a separate population of Rab1-GDP via cholination [33–36].

An even more elegant form of effector regulation was recently described, wherein the effector SidH was inactivated by LubX, a *L*. *pneumophila* secreted E3 ubiquitin ligase that marks SidH for proteasome-dependent proteolysis by polyubiquitination [25]. The key to effector regulation in this case was the differential translocation of LubX and SidH into host cells, with SidH being rapidly secreted followed by the slower intracellular accumulation of LubX. Based on these results, LubX was described as being a “metaeffector”, which was defined as an effector that regulates another effector protein [25]. Reminiscent of the differential regulation and secretion described for SidH and LubX, the expression of SidE proteins is induced in early stationary phase allowing export to occur immediately upon host cell infection [16]. In contrast, SidJ is expressed constitutively [18] and accumulates within the host cell at later time points of infection (Fig. 4). The gradual accumulation of intracellular SidJ during infection correlates with the decreased level of the SidE proteins on the LCV.

**Fig 7:**
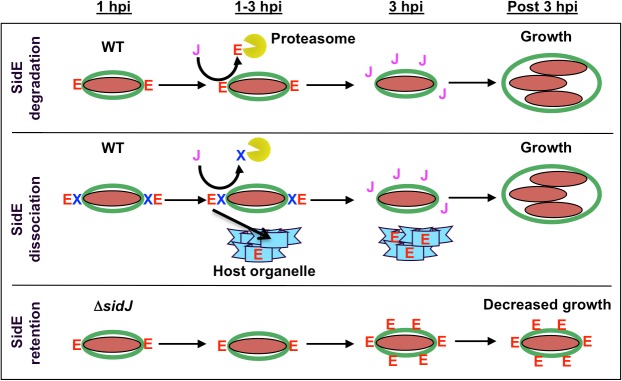
Model showing SidJ-mediated removal of SidE proteins from the *Legionella* containing vacuole (LCV). Shown is a time course for the initial hours of an infection by wild-type *L*. *pneumophila* and a Δ*sidJ* mutant. SidE proteins are indicated with a red letter E, SidJ protein with a purple letter J, a hypothetical protein necessary for retaining SidE proteins on the LCV with a blue letter X, the LCV membrane in green, *L*. *pneumophila* in maroon, the proteasome as a yellow Pac-man, and a host organelle in light blue. In a wild-type infection, SidE proteins localize to the LCV at early time points (1 hpi). At later time points, SidJ removes SidE proteins from the LCV either by localized degradation of SidE (top panel) and/or by degradation of a retention factor, thus leading to the relocalization of LCV-associated SidE proteins to a host organelle (middle panel). In a Δ*sidJ* infection, SidE proteins remain and accumulate on the LCV, which is detrimental for growth (lower panel).

These observations prompted us to propose a model whereby SidJ functions as a metaeffector to modulate the activity of the SidE proteins (Fig. 7). In this model, SidE proteins are translocated into the host cells by the *L*. *pneumophila* Dot/Icm T4SS at early points of infection and localize on the cytoplasmic face of the immature LCV. Although the precise molecular function of the SidE proteins is not yet known, their early delivery into the host cell suggests they are involved in avoidance of the endocytic pathway and/or maturation of the LCV. As the infection proceeds, the SidJ protein begins to accumulate in the host cell, eventually reaching a critical threshold when it is competent to mediate the removal of the SidE proteins from the LCV (Fig. 7). Based on the inhibition by MG132, the simplest possibility is that SidJ directly targets SidE proteins for degradation by the proteasome (Fig. 7, top row). Alternatively, it is possible that SidJ mediates the degradation of another component that normally retains SidE proteins on the LCV surface. In the absence of this factor, the SidE proteins would no longer associate with the LCV and thus could redistribute and potentially associate with host organelles (Fig. 7, middle row).

In the absence of SidJ, SidE proteins appear to localize normally to the LCV at early time points of infection (Fig. 7, bottom row). However, as the infection proceeds, the SidE proteins are no longer removed from the LCV, they accumulate to high levels, eventually inhibiting the growth of *L*. *pneumophila*. Overproduction of SdeA in the absence of SidJ was toxic to both yeast cells and HEK293 cells and inhibited the growth of *L*. *pneumophila* due to delivery of the LCV to the lysosome. The disruption of the Golgi in mCherry-SdeA transfected cells suggests that the target of SdeA is likely to be a component of the secretory pathway.

The failure to eliminate SidE proteins from the LCV in a Δ*sidJ* mutant does not appear to be due to an indirect effect of the LCV not maturing as we can induce removal of SidE proteins from 4-hour LCVs *in vitro* by the addition of wild type SidJ. Rather we prefer the idea that SidJ directly mediates removal of SidE proteins from the LCV, perhaps by some form of post-translational modification. Although we have been unable to reproducibly demonstrate a robust interaction between SidJ and SdeA, it is reasonable that the proteins interact based on the SidJ suppression of SdeA-mediated toxicity in yeast and HEK293 cells. It is also possible that SidJ, which is a large protein of ∼90 kDa, possesses multiple biochemical activities, particularly since a partial ER recruitment defect has been reported for a Δ*sidJ* mutant [18].

In summary, the Dot/Icm substrate SidJ functions as a metaeffector to regulate the activity of the SidE substrates. Similar to the metaeffector LubX, SidJ promotes the removal of Dot/Icm T4SS effectors from the LCV in a proteasome-dependent manner. The presence of dual effectors with opposing activities, and the existence of metaeffectors that modulate the activity of other effectors, may partially explain why *L*. *pneumophila* translocates such a vast repertoire of T4SS substrates into host cells. Moreover, the discovery of a second metaeffector in *L*. *pneumophila* suggests that the concept of metaeffectors is not unique to LubX and additional pathogens may use similar strategies to highjack host cells.

## Materials and Methods

### Ethics statement

This study was carried out in strict accordance with the recommendations in the Guide for the Care and Use of Laboratory Animals of the National Institutes of Health. Protocol 20120081 was approved by the Institutional Animal Care and Use Committee at the Washington University School of Medicine. All efforts were made to minimize suffering.

### Bacterial strains, plasmids, media, and cell lines

Bacterial strains, plasmids, and primers are listed in S1 Table. Detailed plasmid construction is described in S2 Table. All *L*. *pneumophila* strains were cultured on ACES [*N*-(2-acetamido)-2-aminoethanesulfonic acid]-buffered charcoal yeast extract agar (CYE) or in ACES-buffered yeast extract broth (AYE) [37]. Antibiotics and thymidine (100 μg/ml) were added as needed. Strain Lp02 (*thyA hsdR rpsL*) is a derivative of the clinical isolate *L*. *pneumophila* Philadelphia-1 [38]. *E*. *coli* strain XL1 Blue was grown in Luria-Bertani (LB) broth or on LB agar with antibiotics as needed. Yeast strains were grown in YPD medium or yeast minimal medium supplemented with amino acids as needed. A/J mice were obtained from Jackson Laboratories. Mouse bone marrow-derived macrophages (BMMs) were differentiated from stem cells isolated from the femurs of female A/J mice and cultured in RPMI-1640 containing 20% FBS, 1.6 mM glutamine, 30% L-cell culture medium, and penicillin (10,000 IU/ml)/streptomycin (10 mg/ml) for one week as previously described [6,39]. *Acanthamoeba castellanii* cultures were maintained in PYG broth as previously described [40]. HEK-293 cells (obtained from American Type Culture Collection, Manassas, VA) were maintained in DMEM supplemented with 10% heat-inactivated fetal calf serum (FBS) (HyClone, Logan, UT) in a humidified CO_2_ incubator at 5% CO2 concentration. Human monocytic cell line U937 [41] were cultured in RPMI-1640 supplemented with 10% FBS and 2 mM glutamine. To differentiate the cells, they were treated with phorbol 12-myristate 13-acetate (Sigma, St. Louis, MO) for 36 h before use.

### Intracellular growth assay

Intracellular growth of *L*. *pneumophila* was assayed using *A*. *castellanii* as a host cell. *A*. *castellanii* was propagated using PYG medium. Cells were grown to near confluency, recovered, counted, and plated into 24-well culture dishes at a density of 6 x 10^5^ per well. The following day, the cells were washed and equilibrated at 37°C for 1 hour in *A*. *castellanii* buffer [40,42–44]. The amoebae were infected with stationary phase *L*. *pneumophila* cells at a multiplicity of infection (MOI) of 0.2 for 1 hour, washed three times to remove extracellular bacteria, and incubated for two days. *L*. *pneumophila* growth was assayed at 0, 20, 32, and 44 hours. At each time point, infected amoebae were lysed with 0.05% saponin (Sigma, St. Louis, MO) in PBS, the lysate was serially diluted and plated on CYE plates to assess bacterial growth. All growth assays were performed in triplicate.

### Expression of *L*. *pneumophila* proteins in yeast

*Legionella sidJ* and *sdeA* ORFs were cloned into the yeast expression vectors under control of the Pgal or Pcyc promoters (S1 and S2 Table). Plasmids were transformed into yeast cells (JY221, S1 Table) using the lithium acetate/PEG method [45]. Transformed cells were plated on yeast minimal media (US Biological, Massachusetts, MA), synthetic complete (SC) media lacking uracil (Ura) or leucine (Leu) in order to select for transformants. To determine the effect of SidJ or SdeA protein on yeast cell growth, strains were grown to saturation in SC minus Ura or SC minus Leu media containing 2% glucose. Cells were then adjusted to an A600 of 1.0, serially diluted 10-fold, and 5 μl of each dilution was spotted onto SC minus Ura or SC minus Leu containing either 2% glucose (non-induction) or 2% galactose (induction). Plates were incubated at 30° C for 48–72 hr and growth of recombinant strains was recorded.

### Mammalian cell transfections and immunofluorescence

For transfection of YFP-SidJ and mCherry-SdeA, HEK293 cells were seeded onto glass coverslips in 24-well dishes, incubated for one day, then transfected with 0.2 μg plasmid DNA using FuGene6 (Invitrogen, Grand Island, NY) as described by the manufacturer. Transfections were allowed to proceed for 20–40 hr in DMEM supplemented with 10% FBS at 37°C with 5% CO2. Cells were then fixed with 4% paraformaldehyde for 20 min at room temperature, and fixed cells were analyzed by fluorescence microscopy. For the Golgi fragmentation assay, cells were permeabilized with 100% methanol for 10 sec, and then blocked for 10 min with 5% goat serum in PBS. Cells were then stained with anti-giantin antibody (1:400, Covance, Princeton, NJ), followed by Alexa blue-conjugated goat anti-rabbit IgG (Invitrogen, Grand Island, NY) as a secondary antibody. Coverslips were mounted using ProLong Gold antifade reagent (Invitrogen, Grand Island, NY) before examined by fluorescence microscopy.

### Effector protein secretion assays using adenylate cyclase reporter

To quantitate effector protein secretion, we measured the adenylate cyclase activity of CyaA fusions (S1 Table). Differentiated U937 cells were plated into 24-well tissue culture plates at 2.5 x 10^6^ per well. *Legionella* cultures, induced with IPTG at mid-log phase and grown two more hours to reach stationary phase, were harvested, washed, and diluted in RPMI-1640 supplemented with 10% FBS. 5 x 10^6^ bacteria were added to each well for 1 hour, followed by washing three times with cold PBS to remove non-adherent cells, and lysis in 200 μl of lysis buffer (50 mM HCl and 0.1% Triton X-100) on ice. The lysates were transferred to 1.5 ml tubes, boiled for 5 min, and 12 μl of 0.5 M NaOH was added to neutralize the samples. cAMP was extracted using 2 volumes of 95% ethanol and collected after centrifugation at 12,000 g for 5 min to remove cell debris and then lyophilized. Total cAMP concentration was measured using an ELISA kit (GE Healthcare, Pittsburgh, PA).

### Western blot analysis

Protein samples were collected and boiled for five minutes in Laemmli sample buffer and separated by SDS-PAGE gel electrophoresis, followed by transfer to PVDF membranes [46,47]. Membranes were blocked in BLOTTO (PBS containing 5% non-fat dry milk), washed with wash buffer (PBS containing 0.05% Tween 20) and incubated for 1 hour with antibody diluted in BLOTTO. Blots were washed with wash buffer followed by one hour incubation with secondary goat anti-rabbit antibody conjugated to horseradish peroxidase (Sigma, St. Louis, MO) diluted 1:10,000 in BLOTTO. Blots were subsequently washed with wash buffer prior to development using an ECL detection kit (GE Healthcare, Pittsburgh, PA) according to their protocol.

### Immunofluoresence assay

Mouse BMM were seeded on glass coverslips at 1 x 10^5^ cells in 24-well plates and incubated overnight. *Legionella* cells were grown to stationary phase in AYE, washed in sterile water, then adjusted to OD600 = 1.0. Cells were diluted in warmed RPMI-1640 and 5 x 10^5^ bacteria were added to wells containing BMM attached to coverslip. BMMs were infected for 1 hour. After washing to remove uninfected bacteria, cells were fixed using Periodate-Lysine-Paraformaldehyde (PLP) [48] and then permeabilized with methanol for 10 seconds. For effector localization, cells were stained with the SidE family antibody that was raised against SdeC (1:1,000) [16], LidA (1:1,000) [31], or SidM (1:300) [49] antibodies followed by goat anti-rabbit secondary antibody conjugated to Oregon Green (1:1:1,000) (Molecular Probes, Eugene, OR). DNA (bacteria and host nuclei) was stained with propidium iodide (1 mg/ml, Invitrogen, Grand Island, NY). Coverslips were mounted using ProLong Gold antifade reagent (Invitrogen, Grand Island, NY) before being examined by fluorescence microscopy. *Legionella* containing vacuoles (LCVs) decorated with effectors were scored positive by the visual presence of foci of Oregon Green adjacent to bacterial-shaped propidium-iodide staining.

### Fractionation of secreted SidJ and SidE family effectors

To detect the intracellular localization of SidJ and SdeA, differentiated U937 cells were plated at a density of 1 x 10^7^ cells per well in a 6-well plate. The next day, U937 cells were infected for 1 hour with stationary phase cultures of *L*. *pneumophila* and washed three times with PBS to remove uninfected bacteria. Cells were harvested using a cell scraper, washed once with cold PBS and pelleted. To fractionate the lysates, harvested cells were dounced in cold PBS without digitonin. Unbroken cells were removed by centrifugation (3 min, 200 *g*) at 4°C. Pellets and supernatant were separated by centrifugation at 12,000 *g* for 10 min to collect the pellet and cytosolic fraction. To fractionate secreted effector proteins, cells were resuspended in lysis buffer (PBS containing 0.2% digitonin) and dounced. Unbroken cells were then removed by centrifugation (3 min, 200 *g*) at 4°C. The secreted effector proteins were collected from supernatant after removing cell pellets by centrifugation (10 min, 12,000 *g*). Samples were analyzed by Western blot with SidE, SidJ, DotF, LidA, and SidM specific antibodies as above.

### Separation of *Legionella* containing vacuoles (LCV)

Separation of LCV by sucrose density gradient ultracentrifugation was performed as previously described [50]. Postnuclear supernatant (PNS) of infected cells was prepared as follows. Briefly, 1 x 10^7^ differentiated U937 cells plated in 6-well plate were infected with stationary phase *L*. *pneumophila* at an MOI of 5. At the indicated time, the infected cells were suspended in 2 ml of homogenization buffer (20 mM HEPES pH 7.2, 250 mM sucrose, 0.5 mM EGTA) and were gently disrupted in a 7-ml dounce homogenizer. Unbroken cells and nuclei were pelleted by centrifugation at 4°C (3 min, 200 *g*). The PNS containing the *L*. *pneumophila* vacuoles were layered onto a 25–65% sucrose gradient and centrifuged at 100,000 *g* for 1 hour at 4°C. Fractions were collected from the bottom of the gradients and analyzed by SDS-PAGE followed by Western blotting. Separation of LCV from cell organelles was assessed by monitoring for the presence of DotF, a component of the Dot/Icm T4SS.

### Protein purification and *in vitro* SidE family dissociation assay

The *sidJ* open reading frame was amplified and inserted into pQE-30 to express His-SidJ (S1 and S2 Table). *E*. *coli* strain XL1Blue, harboring the resulting plasmid, pJB5331, was used to purify His-tagged SidJ with Ni-NTA columns according to protocols suggested by the manufacturer (Qiagen, Valencia, CA). Dissociation of SidE proteins from PNS was performed as below. Briefly, 2 x 10^6^ BMM cells plated in 6 well-plate were infected with stationary phase *L*. *pneumophila* at an MOI of 0.5, incubated for 1 hour, followed by washing with warm RPMI-1640 to remove uninfected bacteria. At the indicated time, the infected cells were suspended in two ml of homogenization buffer containing 250 mM sucrose in PBS and dounced with 3 strokes. PNS was collected by removing unbroken cells by centrifugation at 4°C (3 min, 200 *g*). Collected PNS were incubated with 0.5 μM of purified SidJ for 30 min at room temperature. The reaction was stopped by addition of equal volume of 4% paraformaldehyde in a stock PLP solution. The fixed cells were attached on lysine-coated glass slides and dissociation of SidE family from LCV was monitored by immunofluorescence detection using SidE antibody (1:1,000 dilution) or LidA (1:1,100 dilution), followed by goat anti-rabbit Oregon Green secondary antibody (Molecular Probes, Eugene, OR). DNA was stained with DAPI and coverslips were mounted using ProLong Gold antifade reagent (Invitrogen) before being examined by fluorescence microscopy. *Legionella* containing vacuoles (LCVs) decorated with effectors were scored positive by the visual presence of foci of Oregon Green adjacent to bacterial-shaped DAPI staining.

### Statistical analysis

Statistical analysis was performed using the GLIMMIX procedure of SAS 9.4 (9.4 SAS Institute Inc.). Data are presented as means ± SEM from three independent experiments. Statistical significance was declared if *P*<0.05.

## Supporting Information

S1 Fig Relationship between SidE family members (SidE, SdeA, SdeB, SdeC) and SidJ. (A) Proteins were analyzed in a two-way comparison using BlastP. Shown is the percent identity between the proteins. SidE family members share extensive homology to each other but are distinct from SidJ. (B) Alignment and phylogenetic tree of the SidE proteins by Clustal analysis.
(TIF)

S2 Fig Overexpression of SdeA inhibits intracellular growth of the *L*. *pneumophila* SuperΔP170. Intracellular growth of *L*. *pneumophila* strains was assayed in *A*. *castellanii* at the indicated time points post infection and replication was expressed as fold growth. Shown are JV1139 (wild-type Lp02 + vector, filled squares), JV4444 (SuperΔP170 + *sdeA*, open circles), JV6756 (SuperΔP170 + *sidJ*, stars), JV3991 (SuperΔP170 *+* vector, open inverted triangles), JV4451 (SuperΔP170 + SdeA overproduction, x’s), and JV1141 (T4SS-deficient *dotA* Lp03 + vector, open squares).
(TIF)

S3 Fig Expression of SidE family proteins and SidJ in various *L*. *pneumophila* strains. Proteins were analyzed in the wild-type strain Lp02, a Δ*sidJ* mutant, the CleanΔP170 mutant, the SuperΔP170 mutant, and the latter two deletion strains expressing wild type levels of SdeA or over producing SdeA (OP). Strains were grown to early stationary phase, harvested and westerns were performed using an antibody to SidE family proteins, SidJ or the constitutively expressed housekeeping protein ICDH. The SidE antibody, originally described in Bardill *et al* 2005, was raised against SdeC and recognizes SdeC, SdeB, and SdeA (SidE cannot be detected and therefore is believed to not be expressed under these conditions). Although the SidE antibody recognizes SdeC, SdeB, and SdeA, it is not clear how efficiently it recognizes each protein, i.e. it is not possible to compare relevant amounts of the three proteins in the westerns. The following results can be observed in this figure: (1) SidE proteins (SdeC, SdeB, SdeA) cannot be detected in the CleanΔP170 mutant or the SuperΔP170 mutant but are normally expressed in the Δ*sidJ* mutant. (2) SidJ cannot be detected in the SuperΔP170 or the Δ*sidJ* mutant but is expressed in the CleanΔP170 mutant. (3) The amount of SdeA normally expressed in a wild-type strain can be observed in the Δ*sdeCB* double deletion (lane 5). (4) pJB3356, the original *sdeA* complementing clone used in Bardill *et al*, expresses wild-type levels of SdeA in both the CleanΔP170 mutant (lane 6) and the SuperΔP170 mutant (lane 7). (5) In contrast, pJB3543 (the over producing SdeA clone) expresses significantly higher amounts of SdeA in both the CleanΔP170 mutant (lane 8) and the SuperΔP170 mutant (lane 9).
(TIF)

S4 Fig There is no secretion dependence between SidJ and the SidE protein SdeA. U937 cells were infected for 1 hour with strains expressing either CyaA alone or SdeA or SidJ fused to CyaA in the indicated strains. Export was measured by the production of cAMP/well. The levels of cAMP are the means ± SEM obtained from an experiment performed in triplicate. The following strains were used: JV6482 (Lp02 + CyaA), JV2700 (Lp02 + CyaA-SdeA), JV3908 (Lp03 + Cya-SdeA), JV3957 (Δ*icmS* + Cya-SdeA), JV6411 (Δ*sidJ* + Cya-SdeA), JV6702 (Lp02 + CyaA-SidJ), JV6736 (Lp03 + CyaA-SidJ), JV6704 (Δ*icmS* + CyaA-SidJ), and JV6773 (CleanΔP170 + CyaA-SidJ).
(TIF)

S5 Fig Toxicity at late time points during transfection of host cells with mCherry-SdeA. HEK293 cells were transfected with mCherry-SdeA and YFP for 40 hours. In the phase image, cells that are rounding up can be observed (arrows) that also express large dense foci of mCherry-SdeA (red).
(TIF)

S6 Fig The SidJ DD mutant fails to complement the growth defect of a Δ*sidJ* mutant. (A) ClustalW Guide Tree showing the relationship between five versions of SidJ from *L*. *pneumophila* strains, SidJ from one *L*. *longbeachae* strain, and SdjA from *L*. *pneumophila* Philadelphia-I. (B) Clustal alignment of the SidJ homologs. The SidJ DD mutant contains two mutations (D542A D545A) that are conserved in SidJ proteins (indicated with red asterisks). (C) Growth within *A*. *castellanii* was assayed for the following strains: JV1139 (Lp02 + vector, filled squares), JV6755 (Δ*sidJ* + *sidJ*, stars), JV4925 (Δ*sidJ* + vector, inverted open triangles), JV6872 (ΔsidJ + *sidJ* DD, x’s) and JV1141 (Lp03 + vector, open squares). (D) *In vivo* demonstration that the SidJ DD mutant does not remove SidE proteins from the LCV. BMMs were infected with wild-type *L*. *pneumophila* (WT) or JV6872 (Δ*sidJ* + *sidJ* DD) for 0 or 3 h. Infected cells were fixed, stained with anti-SidE antibody and the number of SidE positive-phagosomes were counted. Approximately 75 LCVs were scored and error bars represent means ± SEM of three independent experiments.
(TIF)

S1 Table Strains, plasmids, and primers employed in this study. (PDF)

S2 Table Construction of plasmids employed in this study. (PDF)
