# Supplementary figures and images for: Spatiotemporal Regulation of a Legionella pneumophila T4SS Substrate by the Metaeffector SidJ
Source: PLoS Pathog. 2015 Mar 16;11(3):e1004695. doi: 10.1371/journal.ppat.1004695 (PMC4361747; doi:10.1371/journal.ppat.1004695)

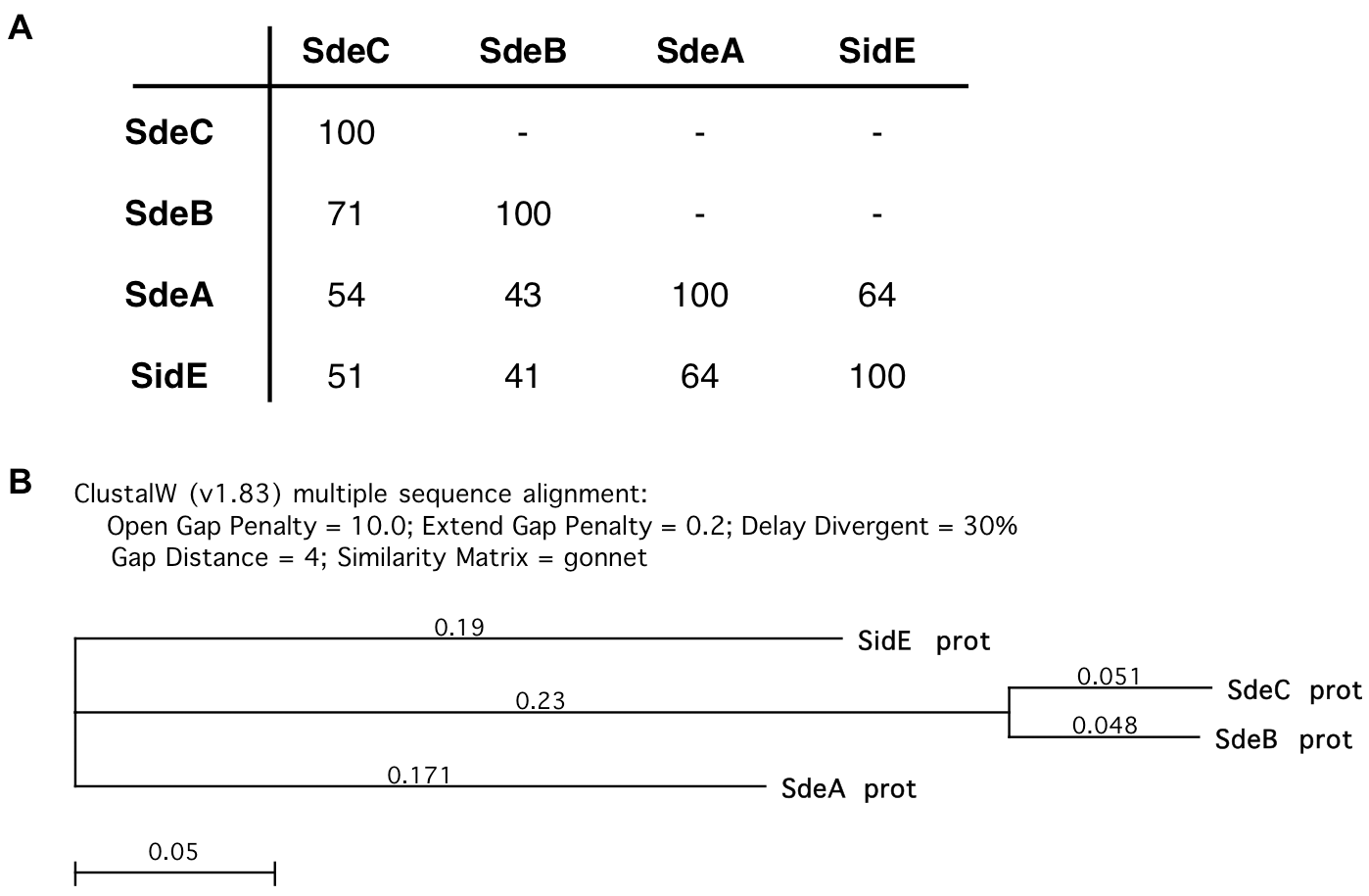

Supplement: S1 Fig — (A) Proteins were analyzed in a two-way comparison using BlastP. Shown is the percent identity between the proteins. SidE family members share extensive homology to each other but are distinct from SidJ. (B) Alignment and phylogenetic tree of the SidE proteins by Clustal analysis. (TIF) [file ppat.1004695.s001.tif]

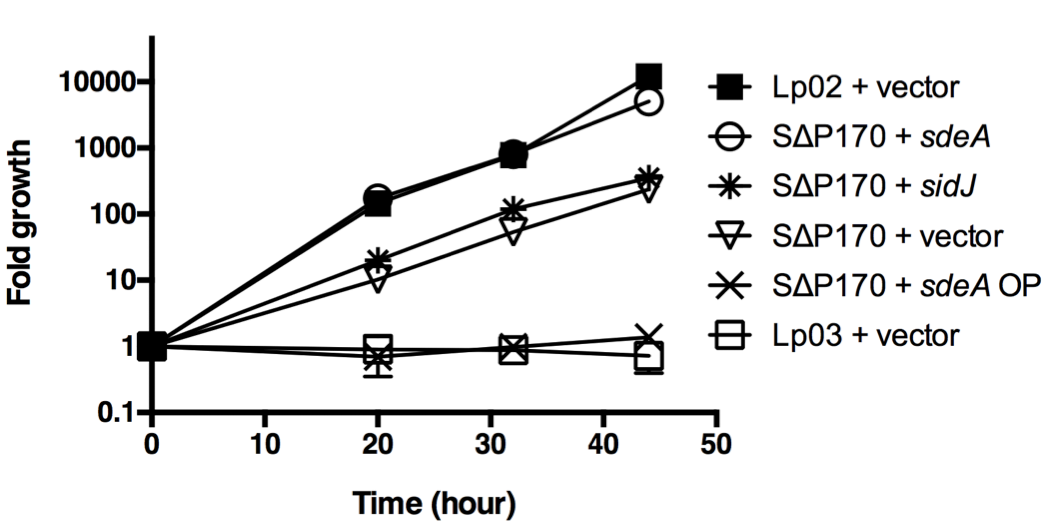

Supplement: S2 Fig — Intracellular growth of L. pneumophila strains was assayed in A. castellanii at the indicated time points post infection and replication was expressed as fold growth. Shown are JV1139 (wild-type Lp02 + vector, filled squares), JV4444 (SuperΔP170 + sdeA, open circles), JV6756 (SuperΔP170 + sidJ, stars), JV3991 (SuperΔP170 + vector, open inverted triangles), JV4451 (SuperΔP170 + SdeA overproduction, x’s), and JV1141 (T4SS-deficient dotA Lp03 + vector, open squares). (TIF) [file ppat.1004695.s002.tif]

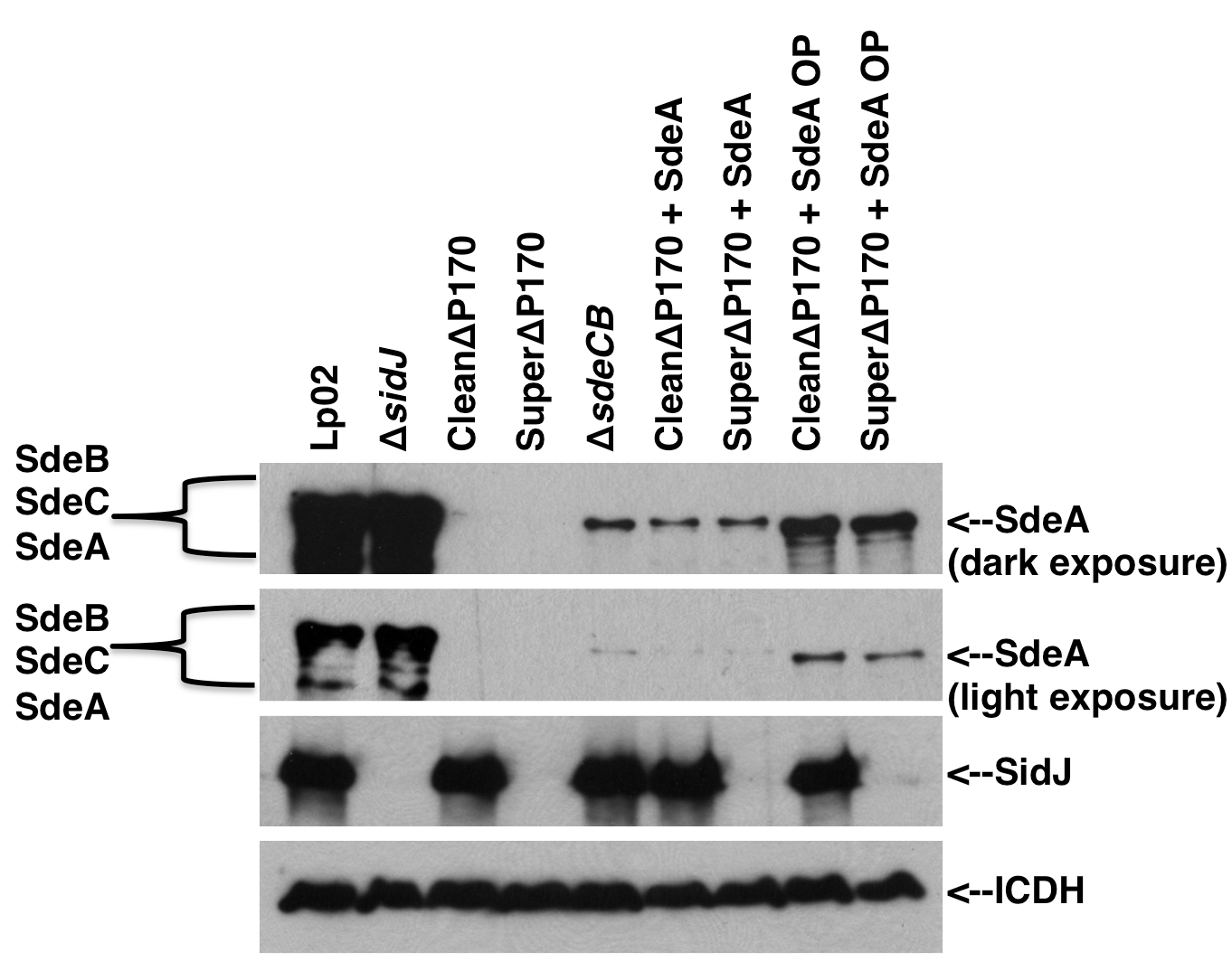

Supplement: S3 Fig — Proteins were analyzed in the wild-type strain Lp02, a ΔsidJ mutant, the CleanΔP170 mutant, the SuperΔP170 mutant, and the latter two deletion strains expressing wild type levels of SdeA or over producing SdeA (OP). Strains were grown to early stationary phase, harvested and westerns were performed using an antibody to SidE family proteins, SidJ or the constitutively expressed housekeeping protein ICDH. The SidE antibody, originally described in Bardill et al 2005, was raised against SdeC and recognizes SdeC, SdeB, and SdeA (SidE cannot be detected and therefore is believed to not be expressed under these conditions). Although the SidE antibody recognizes SdeC, SdeB, and SdeA, it is not clear how efficiently it recognizes each protein, i.e. it is not possible to compare relevant amounts of the three proteins in the westerns. The following results can be observed in this figure: (1) SidE proteins (SdeC, SdeB, SdeA) cannot be detected in the CleanΔP170 mutant or the SuperΔP170 mutant but are normally expressed in the ΔsidJ mutant. (2) SidJ cannot be detected in the SuperΔP170 or the ΔsidJ mutant but is expressed in the CleanΔP170 mutant. (3) The amount of SdeA normally expressed in a wild-type strain can be observed in the ΔsdeCB double deletion (lane 5). (4) pJB3356, the original sdeA complementing clone used in Bardill et al, expresses wild-type levels of SdeA in both the CleanΔP170 mutant (lane 6) and the SuperΔP170 mutant (lane 7). (5) In contrast, pJB3543 (the over producing SdeA clone) expresses significantly higher amounts of SdeA in both the CleanΔP170 mutant (lane 8) and the SuperΔP170 mutant (lane 9). (TIF) [file ppat.1004695.s003.tif]

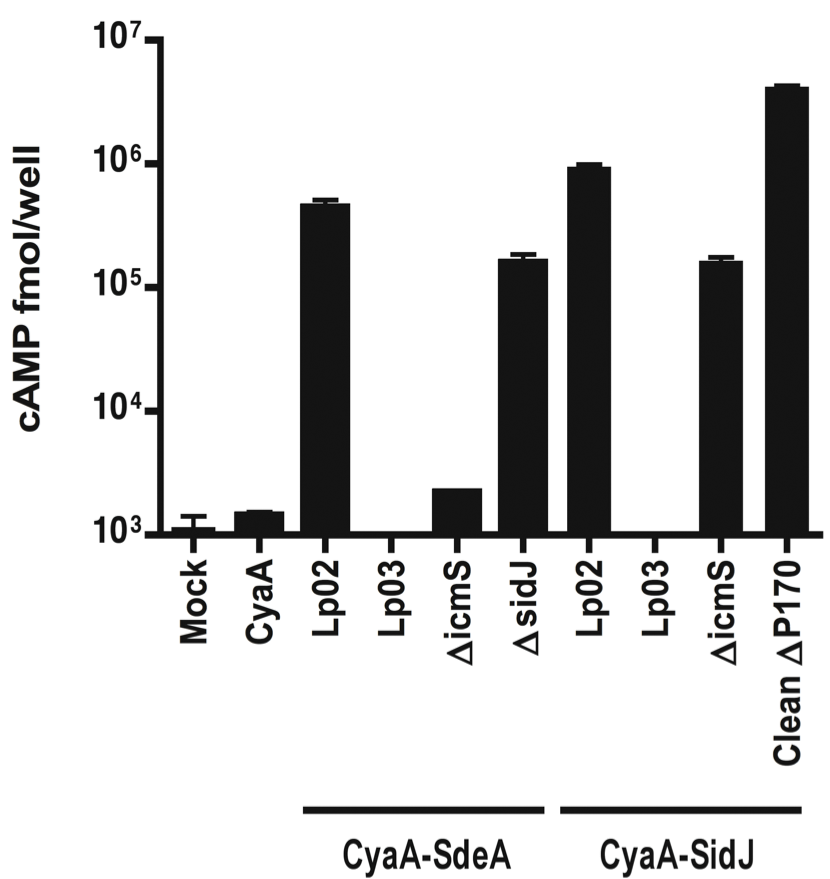

Supplement: S4 Fig — U937 cells were infected for 1 hour with strains expressing either CyaA alone or SdeA or SidJ fused to CyaA in the indicated strains. Export was measured by the production of cAMP/well. The levels of cAMP are the means ± SEM obtained from an experiment performed in triplicate. The following strains were used: JV6482 (Lp02 + CyaA), JV2700 (Lp02 + CyaA-SdeA), JV3908 (Lp03 + Cya-SdeA), JV3957 (ΔicmS + Cya-SdeA), JV6411 (ΔsidJ + Cya-SdeA), JV6702 (Lp02 + CyaA-SidJ), JV6736 (Lp03 + CyaA-SidJ), JV6704 (ΔicmS + CyaA-SidJ), and JV6773 (CleanΔP170 + CyaA-SidJ). (TIF) [file ppat.1004695.s004.tif]

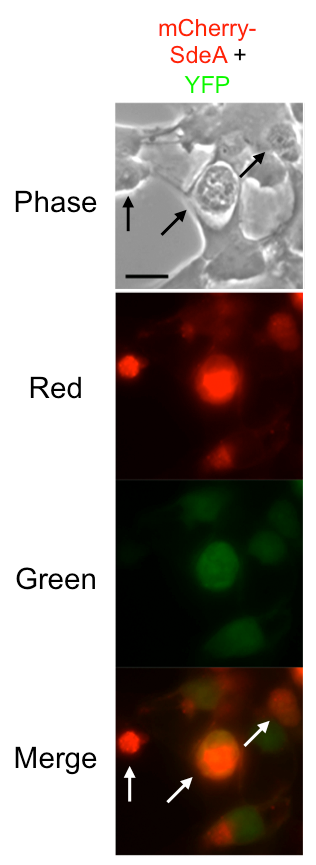

Supplement: S5 Fig — HEK293 cells were transfected with mCherry-SdeA and YFP for 40 hours. In the phase image, cells that are rounding up can be observed (arrows) that also express large dense foci of mCherry-SdeA (red). (TIF) [file ppat.1004695.s005.tif]

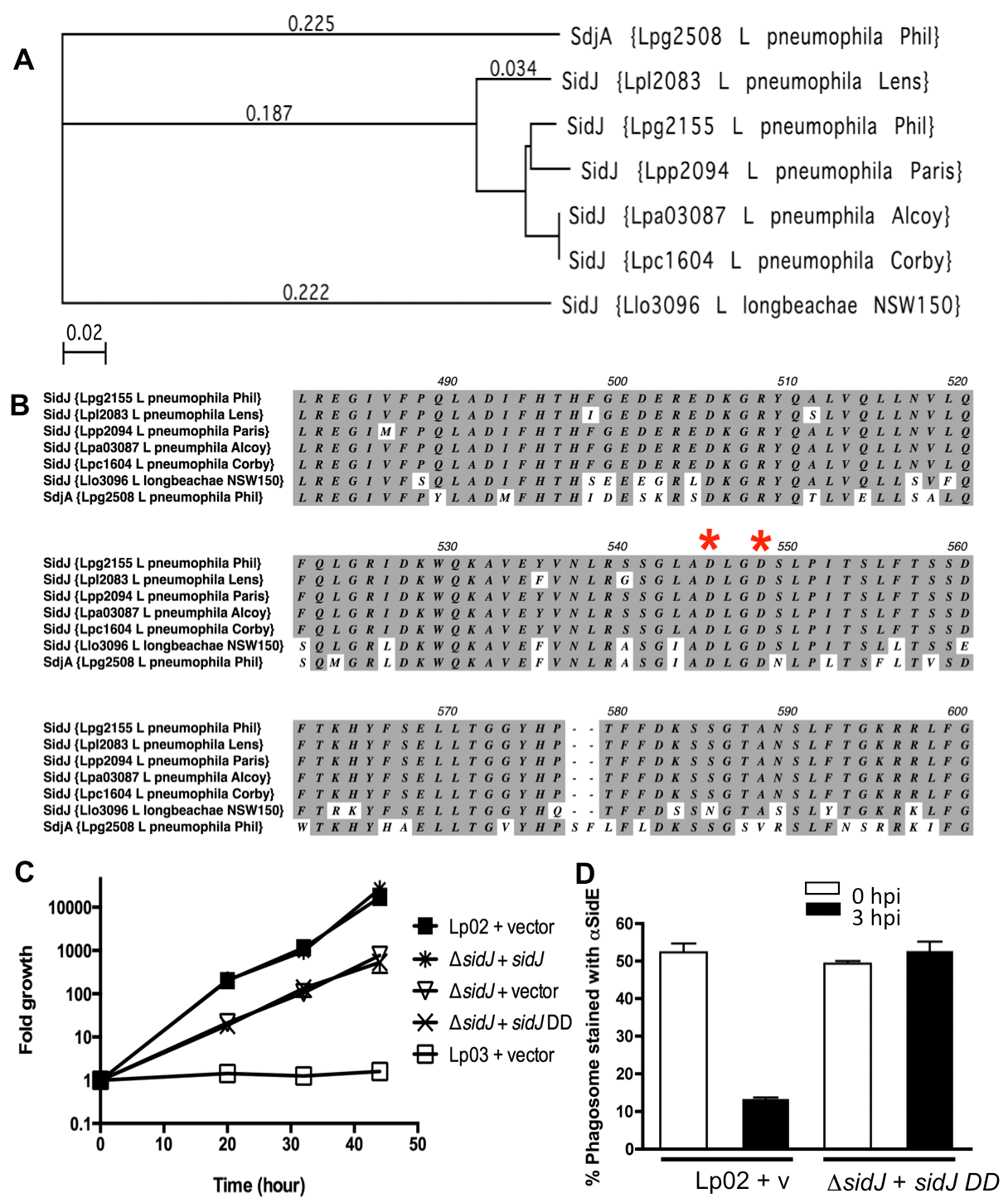

Supplement: S6 Fig — (A) ClustalW Guide Tree showing the relationship between five versions of SidJ from L. pneumophila strains, SidJ from one L. longbeachae strain, and SdjA from L. pneumophila Philadelphia-I. (B) Clustal alignment of the SidJ homologs. The SidJ DD mutant contains two mutations (D542A D545A) that are conserved in SidJ proteins (indicated with red asterisks). (C) Growth within A. castellanii was assayed for the following strains: JV1139 (Lp02 + vector, filled squares), JV6755 (ΔsidJ + sidJ, stars), JV4925 (ΔsidJ + vector, inverted open triangles), JV6872 (ΔsidJ + sidJ DD, x’s) and JV1141 (Lp03 + vector, open squares). (D) In vivo demonstration that the SidJ DD mutant does not remove SidE proteins from the LCV. BMMs were infected with wild-type L. pneumophila (WT) or JV6872 (ΔsidJ + sidJ DD) for 0 or 3 h. Infected cells were fixed, stained with anti-SidE antibody and the number of SidE positive-phagosomes were counted. Approximately 75 LCVs were scored and error bars represent means ± SEM of three independent experiments. (TIF) [file ppat.1004695.s006.tif]
