# Supplementary material for: Spatiotemporal Regulation of a Legionella pneumophila T4SS Substrate by the Metaeffector SidJ
Source: PLoS Pathog. 2015 Mar 16;11(3):e1004695. doi: 10.1371/journal.ppat.1004695 (PMC4361747; doi:10.1371/journal.ppat.1004695)
Supplement: S1 Table — (PDF) [file ppat.1004695.s007.pdf]

Table S1. Strains, plasmids, and primers employed in this study

Strain,plasmid, primer	Relevant properties	Reference or source
Legionella		
Lp02	Wild type *Legionella*	Berger & Isberg, 1993
Lp03	*dotA* mutant	Berger & Isberg, 1993
JV1139	Lp02 + pJB908: wild type + vector	Bardill et al, 2005
JV1141	Lp03 + pJB908: *dotA* mutant + vector	Bardill et al, 2005
JV1962	Δ*icmS*	Vincent et al, 2006
JV2700	Lp02 + pJB2588: Lp02 + CyaA-SdeA	Bardill et al, 2005
JV2975	SuperΔP170: Δ*sdeC* Δ*orf2* Δ*sidJ* Δ*sdeB* Δ*sdeA*	Bardill et al, 2005
JV3991	JV2975 + pJB908: SuperΔP170 + vector	Bardill et al, 2005
JV3908	Lp03 + pJB2588: Lp03 + CyaA-SdeA	Bardill et al, 2005
JV3925	Δ*sdeC* Δ*sdeB*	Bardill et al, 2005
JV3957	Δ*icmS* + pJB2588: Δ*icmS* + CyaA-SdeA	This study
JV4444	JV2975 + pJB3556: SuperΔP170 + *sdeA*	This study
JV4451	JV2975 + pJB3543: SuperΔP170 + *sdeA* overproduction	This study
JV4487	Δ*sidJ*	This study
JV4622	JV3925 + pJB908: Δ*sdeC* Δ*sdeB* + vector	This study
JV4925	JV4487 + pJB908: Δ*sidJ* + vector	This study
JV4928	JV4487 + pJB3556: Δ*sidJ* + *sdeA*	This study
JV5244	Lp02 + pJB3556: Lp02 + *sdeA*	This study
JV5500	JV6113 + pJB908: CleanΔP170 + vector	This study
JV6113	CleanΔP170: Δ*sdeC* Δ*sdeB* Δ*sdeA* Δ*sidE*	This study
JV6407	JV6113 + pJB3556: CleanΔP170 + *sdeA*	This study
JV6411	JV4487 + pJB2588: Δ*sidJ* + CyaA-SdeA	This study
JV6445	Lp02 + pJB3543: Lp02 + *sdeA* overproduction	This study
JV6450	JV6113 + pJB3543: CleanΔP170 + *sdeA* overproduction	This study
JV6451	JV4487 + pJB3543: Δ*sidJ* + *sdeA* overproduction	This study
JV6703	Lp02 + pJB5145: Lp02 + CyaA-SidJ	This study
JV6704	Δ*icmS* + pJB5145: Δ*icmS* + CyaA-SidJ	This study
JV6736	Lp03 + pJB5145: Lp03 + CyaA-SidJ	This study
JV6755	JV4487 + pJB4047: Δ*sidJ* + *sidJ*	This study
JV6756	JV2975 + pJB4047: SuperΔP170 + *sidJ*	This study
JV6757	JV6113 + pJB4047: CleanΔP170 + *sidJ*	This study
JV6773	CleanΔP170 + pJB5145: Δ*icmS* + CyaA-SidJ	This study
JV6482	Lp02 + pJB5104: CyaA:X fusion vector w/ stop codon	This study
JV6872	JV4487 + pJB5346: Δ*sidJ* + SidJ DD	This study
E. coli		
XL1Blue	*recA1 endA1 gyrA96 thi-1 hsdR17 supE44 relA1 lac* [F' *proAB lacI*^{q}*Z*Δ*M15* Tn*10* (Tet^r)]	Stratagene
Yeast		
JY221	Wild type yeast (BY4730), MATα, *leu*Δ0, *met15*Δ0, *ura3*Δ0	Life Technologies
JY232	Pgal vector (pJB2748) in JY221	This study
JY380	Pgal-*sidJ* (pJB4060) in JY221	This study
JY382	Pgal-*sdeA* (pJB3367) in JY221	This study
JY387	Pgal vector + Pcyc vector in JY221	This study
JY388	Pgal vector + Pcyc-*sidJ* in JY221	This study
JY389	Pcyc-*sidJ* + Pgal-*sdeA* in JY221	This study
JY394	Pcyc vector + Pgal-*sdeA* in JY221	This study
Plasmids		
pJB908	RSF1010 vector, thyA$^+$, *bla, mob*	Sexton et al. 2005
pJB1806	pJB908-*td*Δ*i* + Cm^R	Bardill et al, 2005
pJB1172	pQE30	Qiagen
pJB2182	*sdeA* complementing clone intermediate clone	Bardill et al, 2005

pJB2265	*sdeA* complementing clone intermediate clone	Bardill et al, 2005
pJB2559	Pgal-sdeA intermediate clone	
pJB2581	CyaA-X fusion vector	Bardill et al, 2005
pJB2588	CyaA-SdeA	Bardill et al, 2005
pJB2748	Pgal vector (Real name is pBM272)	Johnston & Davis, 1984
pJB2860	HA fusion vector	Lab collection
pJB3238	CyaA-SidJ without stop	Bardill et al, 2005
pJB3365	pJB1806, Amp^{s}	This study
pJB3367	Pgal-*sdeA*	This study
pJB3543	Native promoter *sdeA* complementing clone	This study
pJB3556	PCR *sdeA* complementing clone	Bardill et al, 2005
pJB3593	Pcyc vector (real name is BM4426)	Mumberg et al, 1995
pJB3953	*sidJ* complementing clone intermediate clone	This study
pJB4047	*sidJ* complementing clone	This study
pJB4060	Pgal-*sidJ*	This study
pJB4078	Pcyc-*sidJ*	This study
pJB5104	CyaA-X with stop codon	This study
pJB5139	Cya-SidJ intermediate clone	This study
pJB5145	CyaA-SidJ	This study
pJB5205	CyaA-X w/o HindIII of Cm^{R}	This study
pJB5331	His-SidJ in pQE30	This study
pJB5346	SidJ DD mutant (D542A, D545A)	This study
pJB5604	YFP expression RSF1010	This study
pJB5609	His-SidJ DD mutant in pQE30	This study
pJB5619	YFP-SidJ intermediate	This study
pJB5621	YFP-SdeA intermediate	This study
pJB5687	pcDNA3/1 (+), mammalian expression vector	Life Technologies
pJB5708	YFP-SidJ mammalian expression clone	This study
pJB5710	YFP fusion mammalian expression vector	This study
pJB5774	mCherry fusion mammalian expression vector	This study
pJB5787	mCherry-SdeA Mammalian expression clone	This study
pJB5859	pcDNA6.2/N- N-mCherry-DEST	Life Technologies

Primers

JVP856	GGGGGATCCCCTAAGTATGTCGAAGGGGTAG
JVP857	GGGGTCGACACTTCATGCGATTTGGGTTAAGG
JVP895	CCCGAATTCAGGAGAAATTACTATGCAGCAATCGCATCAGGC
JVP896	CCCGGATTCATAGCCGGAATCCTGGCGTTCC
JVP993	CAAGGCGCACTCCCGTTCTGG
JVP1284	GGCCAAGGCCACCGCGAGACCCGCAGACCAAAACGATCTCAAG
JVP1381	CCCGTCGACTCACAAACGTTTATCAGTAGTACGTTCG
JVP1460	CCCGGATCCAGGAGAAATTACTATGTTTGGTTTCATAAAGAAAGTAC
JVP1897	CGTGCACATTTGTTTTAACAGAAGC
JVP1911	CCCGGATCCTCTAGATAAATATTTGAATTTATGTTTGGTTTCATAAAGAAAGTAC
JVP1934	CCCCTGCAGGCGGCCGCTCAATCGACGGACAATCCAACACC
JVP2005	CCCGGATCCTCTAAAGACCTTGAAGTGTATGTTTATAAAGCCCC
JVP2079	GGCTCCCAAAGCAGCCAGACCACTGCTGCGC
JVP2080	GGTCTGGCTGCTTTGGGAGCCAGTCTGCCCATAACTAGCCTG
JVP2159	CCCTCTAGATCAGGATCCTTTGTATAGTTCATCCATGCC
JVP2261	CCCGGTACCCGCCACCATGGTCTCTAAGGGCGAGGAAGAC
JVP2262	CCCGGATCCTTTGTACAGCTCATCCATGCC
