# Supplementary material for: Spatiotemporal Regulation of a Legionella pneumophila T4SS Substrate by the Metaeffector SidJ
Source: PLoS Pathog. 2015 Mar 16;11(3):e1004695. doi: 10.1371/journal.ppat.1004695 (PMC4361747; doi:10.1371/journal.ppat.1004695)
Supplement: S2 Table — (PDF) [file ppat.1004695.s008.pdf]

Table S2. Construction of plasmids employed in this study

Plasmid	Relevant properties	Vector[a]	Enzymes to digest vector	Primers[b] plasmid[c]	Enzymes to digest insert
pJB2559	Pgal-*sdeA* intermediate	pJB2860	BamHI/SalI	JVP856/ JVP857	BamHI/SalI
pJB3365	pJB1806, Amp^S	pJB1806	HindIII/BsaI	JVP993/ JVP1284	HindIII/BsaI
pJB3367[d]	Pgal-*sdeA*	pJB2748	SphI/SalI	pJB2559	SphI/SalI
pJB3543	*sdeA* complementing clone	pJB2265	KpnI/SnaBI	pJB2182	KpnI/SnaBI
pJB3953	*sidJ* complementing clone intermediate	pJB908	BamHI/SalI	JVP1460/ JVP1381	BamHI/SalI
pJB4047[e]	*sidJ* complementing clone	pJB3953	BamHI/BglII	JVP1911/ JVP1897	BamHI/BglII
pJB4060	Pgal-*sidJ*	pJB2748	BamHI/SalI	pJB3953	BamHI/SalI
pJB4078	Pcyc-*sidJ*	pJB3593	BamHI/SalI	pJB3953	BamHI/SalI
pJB5104	CyaA-X	pJB1806	EcoRI/BamHI	JVP895/ JVP896	EcoRI/BamHI
pJB5139	CyaA-SidJ intermediate	pJB5205	BamHI/SalI	pJB3238	BamHI/SalI
pJB5145	CyaA-SidJ	pJB5139	XhoI/SalI	pJB3953	XhoI/SalI
pJB5205[f]	CyA-X fusion	pJB2581	HindIII		
pJB5331	His-SidJ in pQE30	pJB3213	BglI/SalI	pJB3953	BglI/SalI
pJB5346[g]	SidJ DD mutant	pJB4047	BglII/XhoI	JVP2005/ JVP2079<br>JVP2080/ JVP1934<br>JVP2005/ JVP1934	BglII/XhoI
pJB5604	YFP expression	pJB3365	EcoRI/XbaI	JVP993/ JVP2159	EcoRI/XbaI
pJB5609	His-SidJ DD mutant	JB5331	BglII/Pst1	pJB5346	BglII/Pst1
pJB5708	YFP-SidJ	pJB5687	EcoRI/SalI	pJB5619	EcoRI/SalI
pJB5710	YFP fusion	pJB5687	EcoRI/SalI	pJB5604	EcoRI/SalI
pJB5774	mCherry fusion	pJB5687	KpnI/BamHI	JVP2261/ JVP2262	KpnI/BamHI
pJB5787	mCherry-SdeA	pJB5774	BamHI/XhoI	pJB5621	BamHI/XhoI

[a]See Table S1 for vector references

[b]Primer sequences are listed in Table S1

[c]Plasmids used for subcloning are listed in Table S1

[d]Example of subcloning procedure (pJB3367): *sdeA* fragment was digested with SphI and SalI from pJB2559 and ligated into pJB2748 digested with SphI/SalI

[e]Example of cloning using PCR amplification (pJB4047): *sidJ* fragment was amplified using primers JVP1911 and JVP1897. The PCR product was digested with BamHI and BglII and ligated into pJB3953 digested BamHI and BglII

[f]Detailed description of pJB5205: The 5' CmR HindIII site of pJB2581 was mutated using Klenow. A partial HindIII digest was performed on pJB2581, the fragment was gel isolated, filled in with Klenow to destroy the site, and ligated back together.

[g]Detailed description of pJB5346: Site directed mutagenesis were used to mutate two aspartic acid

residues of SidJ. In the first round PCR, primers JVP2005/JVP2079 and JVP2080/JVP1934, and pJB3953 (*sidJ* complementing clone) were used to amplify about 300 bp and 400 bp PCR fragments, respectively. After PCR purification, two PCR products were used as templates for a second round PCR. Primers JVP2005 and JVP1934 were used to amplify 700 bp PCR fragment. The PCR product was digested with BglII and XhoI and ligated into pJB4047 digested with BglII and XhoI.
Sequencing was done to confirm that sidJ DD mutant encode D542A/D545A.
